# ALS-Associated *FUS* Mutations Result in Compromised *FUS* Alternative Splicing and Autoregulation

**DOI:** 10.1371/journal.pgen.1003895

**Published:** 2013-10-31

**Authors:** Yueqin Zhou, Songyan Liu, Guodong Liu, Arzu Öztürk, Geoffrey G. Hicks

**Affiliations:** 1 Manitoba Institute of Cell Biology, University of Manitoba, Winnipeg, Manitoba, Canada; 2 Department of Biochemistry & Medical Genetics, University of Manitoba, Winnipeg, Manitoba, Canada; 3 Regenerative Medicine Program, University of Manitoba, Winnipeg, Manitoba, Canada; 4 Faculty of Pharmacy, University of Manitoba, Winnipeg, Manitoba, Canada; Centre for Cancer Biology, SA Pathology, Australia

## Abstract

The gene encoding a DNA/RNA binding protein *FUS/TLS* is frequently mutated in amyotrophic lateral sclerosis (ALS). Mutations commonly affect its carboxy-terminal nuclear localization signal, resulting in varying deficiencies of FUS nuclear localization and abnormal cytoplasmic accumulation. Increasing evidence suggests deficiencies in FUS nuclear function may contribute to neuron degeneration. Here we report a novel FUS autoregulatory mechanism and its deficiency in ALS-associated mutants. Using FUS CLIP-seq, we identified significant FUS binding to a highly conserved region of exon 7 and the flanking introns of its own pre-mRNAs. We demonstrated that FUS is a repressor of exon 7 splicing and that the exon 7-skipped splice variant is subject to nonsense-mediated decay (NMD). Overexpression of *FUS* led to the repression of exon 7 splicing and a reduction of endogenous FUS protein. Conversely, the repression of exon 7 was reduced by knockdown of FUS protein, and moreover, it was rescued by expression of EGFP-FUS. This dynamic regulation of alternative splicing describes a novel mechanism of FUS autoregulation. Given that ALS-associated FUS mutants are deficient in nuclear localization, we examined whether cells expressing these mutants would be deficient in repressing exon 7 splicing. We showed that FUS harbouring R521G, R522G or ΔExon15 mutation (minor, moderate or severe cytoplasmic localization, respectively) directly correlated with respectively increasing deficiencies in both exon 7 repression and autoregulation of its own protein levels. These data suggest that compromised FUS autoregulation can directly exacerbate the pathogenic accumulation of cytoplasmic FUS protein in ALS. We showed that exon 7 skipping can be induced by antisense oligonucleotides targeting its flanking splice sites, indicating the potential to alleviate abnormal cytoplasmic FUS accumulation in ALS. Taken together, FUS autoregulation by alternative splicing provides insight into a molecular mechanism by which FUS-regulated pre-mRNA processing can impact a significant number of targets important to neurodegeneration.

## Author Summary

*FUS/TLS* is a frequently mutated gene in amyotrophic lateral sclerosis (ALS). ALS, also known as Lou Gehrig's disease, is characterized by a progressive degeneration of motor neurons. The abnormal cytoplasmic accumulation of mutant FUS protein is a characteristic pathology of ALS; however, recent evidence increasingly suggests deficiencies in FUS nuclear function may also contribute to neurodegeneration in ALS. Here we report a novel autoregulatory mechanism of FUS by alternative splicing and nonsense mediated decay (NMD). We show FUS binds to exon 7 and flanking introns of its own pre-mRNAs. This results in exon skipping, inducing a reading frame shift and subsequent degradation of the splice variants. As such, this mechanism provides a feedback loop that controls the homeostasis of FUS protein levels. This balance is disrupted in ALS-associated FUS mutants, which are deficient in nuclear localization and FUS-dependent alternative splicing. As a result, the abnormal accumulation of mutant FUS protein in ALS neurons goes unchecked and uncontrolled. Our study provides novel insight into the molecular mechanism by which FUS regulates gene expression and new understanding of the role of FUS in disease at the molecular level. This may lead to new potential therapeutic targets for the treatment of ALS.

## Introduction

Amyotrophic lateral sclerosis (ALS) is a neuronal degenerative disorder caused by progressive loss of motor neurons in brain and spinal cord, leading to paralysis and death [1]. *FUS* is a frequently mutated gene in ALS (combining familial and sporadic ALS), in addition to C9ORF72, SOD1 and TDP-43 [1]–[3]. Most ALS-associated *FUS* mutations are within the nuclear localization signal (NLS) in the carboxyl terminus [2], [4], [5], resulting in increased cytoplasmic FUS localization [6], [7]. The abnormal cytoplasmic aggregation of FUS mutants in neuron and glial cells is a pathological hallmark of ALS and some cases of frontotemporal lobar degeneration (FTLD) [8]–[10]. It's noteworthy that there is a correlation between the observed cytoplasmic FUS accumulation and the age of ALS onset, with the more cytoplasmic FUS accumulation the earlier age of disease onset [8], [11]–[13]. Several studies suggest that cytoplasmic accumulation of FUS mutant protein can lead to direct cytoplasmic cytotoxicity or may indirectly result in the loss of FUS function in the nucleus. Studies in yeast models demonstrated that expression of ALS-associated FUS mutants can lead to protein aggregation and cytotoxicity that recapitulate FUS proteinopathy [14]. Investigations in some *Drosophila*, *C. elegans* and rat models showed that expression of ALS-associated FUS mutants can lead to motor neuron dysfunction and neurodegeneration [15]–[17]. However, some *Drosophila* and zebrafish models support that the loss of FUS function can lead to behavioral and structural defects of motor neurons [18], [19]. Exactly how the loss of FUS nuclear function and/or the gain of cytoplasmic cytotoxicity contribute to neurodegeneration at the molecular level is still unknown.

FUS is predominantly a nuclear protein [20], and binds both DNA and RNA [21], [22]. FUS is involved in multiple steps of RNA metabolism including transcription, pre-mRNA splicing and mRNA transport for site specific translation [23]–[25]. The alteration of FUS-regulated RNA processing is a proposed key event in ALS pathogenesis, given that RNA binding proteins and splicing misregulation are linked to neurological diseases [8], [26], [27]. To understand the normal function of FUS in RNA processing, it is essential to identify FUS RNA targets. Recently a large number of FUS RNA targets in various cell lines and neural tissues were identified by CLIP-seq (cross-linking and immunoprecipitation, followed by high-throughput sequencing), a method to purify protein-RNA complexes coupled with deep sequencing [28]–[32]. The challenge now is to begin to understand what the biological significance of FUS-regulated RNA processing is, and how these processes are altered in FUS mutants and may therefore contribute to ALS pathogenesis.

Our CLIP-seq data in HeLa cells show that FUS binding is enriched in introns flanking cassette exons of pre-mRNAs. Among the identified FUS-binding cassette exons and their flanking introns, the most highly enriched target is exon 7 and flanking introns of *FUS* pre-mRNA itself. Here we demonstrate that FUS is a repressor of exon 7 and that the exon 7-skipped splice variants of *FUS* are subject to nonsense-mediated decay (NMD). Overexpression of *FUS* leads to repression of exon 7 splicing and predictably a reduction of endogenous FUS protein levels. Conversely, knockdown of FUS protein reduces the repression of exon 7. Moreover, the reduction of exon 7 repression can be rescued by the expression of EGFP-FUS. Taken together, these studies show that FUS dynamically autoregulates its own protein levels by directly modulating the alternative splicing of exon 7. Furthermore, our data show that ALS-associated FUS mutants are deficient in nuclear localization, exon 7 repression and autoregulation of its own protein levels. We propose that the compromised FUS autoregulation in ALS forms a feed-forward loop, exacerbating the abnormal cytoplasmic FUS accumulation, and as such, provides a molecular mechanism that can potentially contribute to ALS pathogenesis.

## Results

### FUS CLIP-seq identified significant FUS binding to introns flanking cassette exons

**Figure 1:**
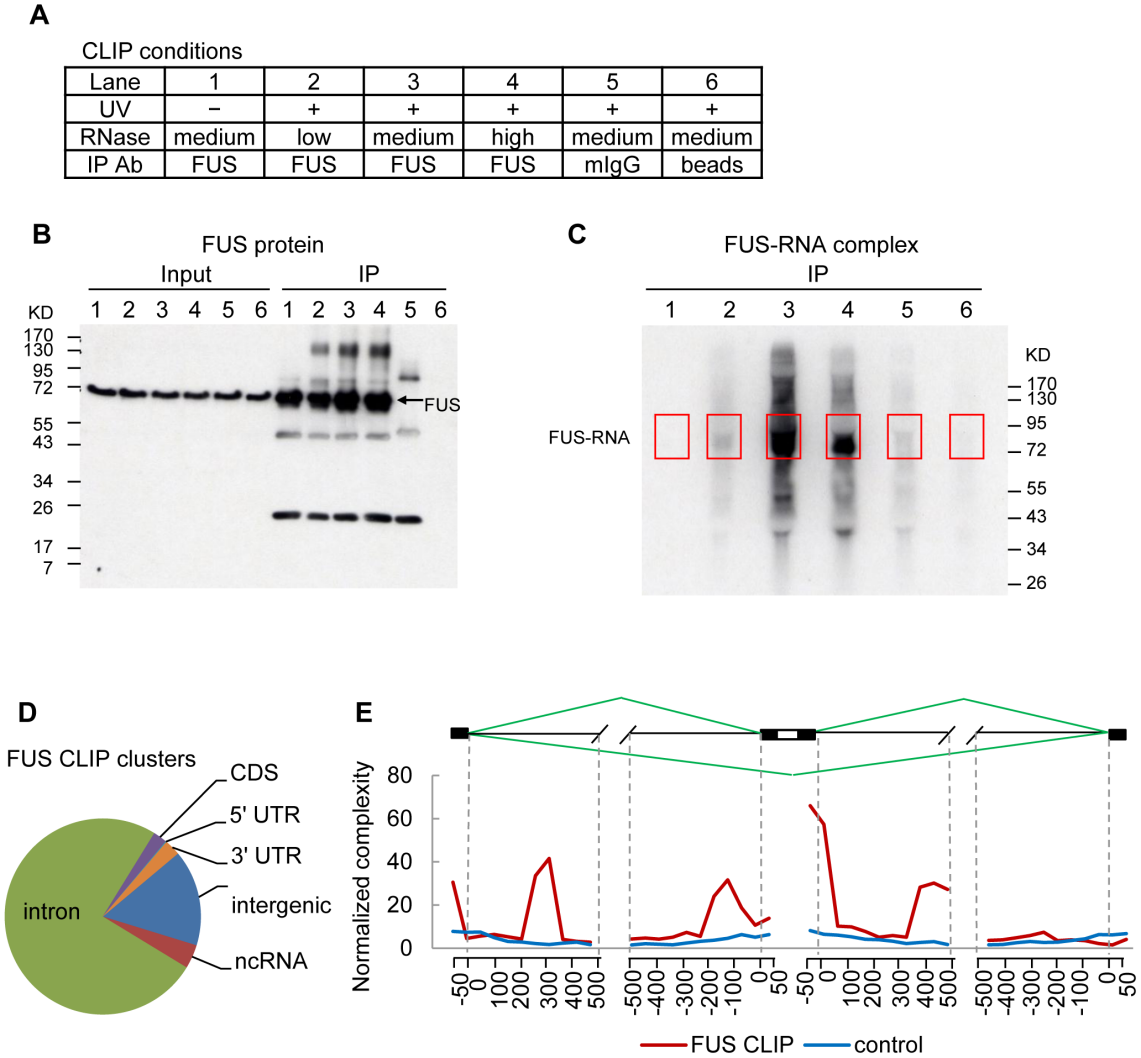
FUS CLIP-seq identified increased FUS binding in introns flanking cassette exons. A) FUS CLIP assay conditions. B) Western blot analysis of FUS protein immunoprecipitated from FUS CLIP. C) Autoradiography of radiolabeled FUS-RNA complexes from FUS CLIP. Red boxes highlight FUS protein complex regions selected for further analysis. D) Percentage of FUS CLIP clusters mapped to the indicated regions (UTR: untranslated region; CDS: coding sequence; ncRNA: non-coding RNA). E) Normalized complexity map of FUS binding at 87 FUS-associated cassette exons and flanking introns. Control was an average of 100 sets of normalized complexity of 87 constitutive exons randomly selected from genes expressed in HeLa cells, as determined by RNA-seq.

To identify RNA targets of FUS, we performed FUS CLIP-seq in HeLa cells. Western blot and autoradiography showed successful immunoprecipitation of FUS protein and FUS-RNA complexes (Figure 1A, 1B and 1C). Sequencing of FUS CLIP RNA yielded 1,879,212 non-redundant reads mapped to human genome GRCh37, with the majority (1,305,507 reads) to pre-mRNAs (Figure S1). Using the peak-finding algorithm CisGenome (www.biostat.jhsph.edu/~hji/cisgenome/) [33], we identified 1928 FUS CLIP clusters (sites containing significantly enriched overlapping FUS CLIP tags) corresponding to 1149 target genes (Table S1) in HeLa cells. FUS RNA targets identified by our CLIP-seq were compared with those previously identified by other CLIP-seq [28]–[30], [32], PAR-CLIP [31] and RIP-chip [34] (Figure S2). HeLa and HEK293 cells [31] share 845 common target genes (Figure S2, Table S2), accounting for 74% of all identified targets in HeLa cells. Gene Ontology (GO) biological process (BP) analysis of these 845 genes showed an enrichment of genes regulating gene expression and transcription. Analysis of various CLIP-seq datasets of mouse brains or neurons [28]–[30], [32] identified 508 common genes, which are enriched for genes regulating cell adhesion, synaptic transmission, glutamate signaling pathways and nervous system development. 120 genes are common to all the datasets analyzed, and show an enrichment of genes regulating cell motion and protein dephosphorylation. Taken together, our analyses revealed cell-type specific and common RNA targets of FUS.

Seventy five percent of our FUS CLIP clusters were located within introns (Figure 1D), consistent with previous reports [28]–[32]. To address the function of FUS in alternative splicing, we analyzed the association between FUS CLIP clusters and known alternative splicing events. Using the UCSC Known AltEvent database as a reference, we scored a FUS CLIP cluster as associated with an alternative splicing event if the CLIP cluster overlapped the alternative splicing event itself or overlapped its immediate flanking introns and/or exons, as described previously [35]. Our analysis identified “cassette exon” as the top category of alternative splicing events associated with FUS CLIP clusters (Figure S3). FUS CLIP clusters are associated with 206 cassette exons in total (Figure S3). To identify FUS binding regions flanking cassette exons, we used 87 FUS-associated cassette exons that are flanked by constitutive exons (Table S3) to generate a normalized complexity map as previously described [36]. We found that FUS binding was enriched in the flanking introns, particularly proximal to splice sites flanking the cassette exons (Figure 1E). The peak at 5′ splice sites, within 100 nucleotides (nt) downstream of the cassette exons, showed the highest enrichment of FUS CLIP tags. The peak proximal to 3′ splice sites was about 150 nt upstream of the cassette exons, instead of immediately upstream of 3′ splice sites (less than 50 nt), as previously described [31]. We also observed a peak at about 400 nt downstream of the cassette exons and a peak at about 300 nt downstream of the upstream constitutive exons. The locations of all these four peaks in our complexity map were also detected as statistically significant FUS binding sites in the complexity map from Lagier-Tourenne *et al*. [30]. Comparing our FUS complexity map with all the other reports [28]–[30], it is consistent that in general FUS binding is enriched in the intronic regions 500 nt upstream or downstream of cassette exons or constitutive exons flanking cassette exons, although the exact nucleotide positions are not identical in different studies. FUS-RNA binding may be both position and sequence dependent.

We next analyzed the sequences of FUS CLIP clusters associated with cassette exons and their flanking introns for possible *de novo* consensus RNA-binding motifs using the HOMER algorithm [37]. Analysis of the CLIP clusters within each individual peak on the complexity map (Figure 1E) did not identify any significant common consensus motifs (data not shown). Individually, the highest FUS-binding peak at the 5′ splice sites downstream of cassette exons did show an enrichment of CAGGUU (2.6 fold, *P* = 0.001) (Figure S4); however, this is expected as CAGGUU is very similar to the human 5′ splice site consensus sequences MAG|GURAGU (M is A or C and R is A or G) [38].

To assess whether genes encoding FUS-associated cassette exons can be clustered into functional groups, we analyzed the Gene Ontology (GO) biological process (BP) terms and KEGG pathways using DAVID Bioinformatics Resources 6.7 [39]. Our results showed that the most enriched GO BP terms were regulation of transcription, RNA metabolic process and neurogenesis (Table S4). The most enriched KEGG pathways were Wnt, adherens junction and Notch signaling pathways (Table S5).

### FUS binds to a highly conserved region spanning exon 7 and the flanking introns of its own pre-mRNA

**Figure 2:**
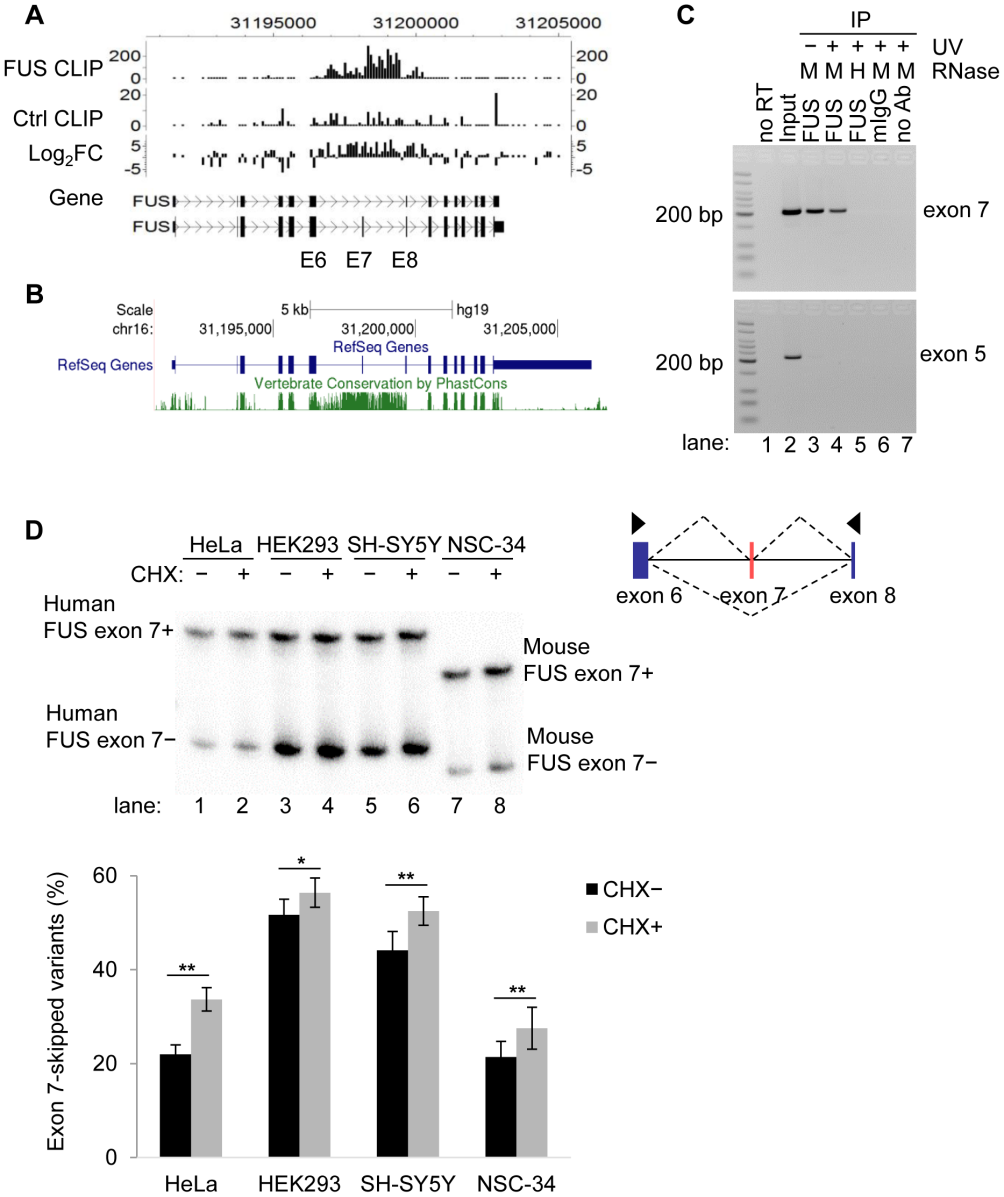
FUS binds to exon 7 and flanking introns of its own pre-mRNA *in vivo*. A) The enrichment of FUS CLIP tags in exon 7 (E7) and the flanking introns of *FUS* own pre-mRNA, as determined by a peak finding algorithm CisGenome. B) Cross-species conservation of *FUS* gene. The conservation track of UCSC genome browser (http://genome.ucsc.edu/) was used to display the PhastCons conservation score of 46 vertebrate species. C) FUS RNA-IP followed by RT-PCR of *FUS* exon 7. RT-PCR of *FUS* constitutive exon 5 is a control. Medium RNase concentration (M; 0.1 µg/ml) or high RNase concentration (H; 1 µg/ml) was used to treat cell lysates before immunoprecipitation. D) *FUS* exon 7-skipped splice variant is subject to nonsense mediated decay (NMD). Cycloheximide (CHX) was used to treat cells for 6 h to inhibit NMD. *FUS* exon 7 splice variants were detected by [γ-^32^P] ATP labeled PCR. The exon skipping ratio is equal to the intensity of the exon 7-skipped band divided by the intensity sum of both splice variants. Bar graphs represent mean ± SEM (n = 5 or 6). For all the quantification, student's *t*-tests were performed. * *P*≤0.05, ** *P*≤0.01.

Out of the 87 cassette exons enriched with FUS CLIP clusters, the top candidate was exon 7 and flanking introns 6 and 7 of the pre-mRNAs of *FUS* itself (Table S3). This CLIP cluster was also in the top 10 of all 1928 FUS CLIP clusters identified, as ranked by fold enrichment. The number of FUS CLIP tags within exon 7 and its flanking introns were 8.1 fold higher than the control mouse IgG CLIP tags (FDR = 0.043), as determined by the peak finding algorithm CisGenome [33] (Figure 2A). The region encompassing *FUS* intron6-exon7-intron7 is ∼3 kb and highly conserved in 38 vertebrate species (Figure 2B). Human and mouse DNA sequences share 77% identity within this region, while the average similarity throughout other introns of *FUS* is 40%. Sub-regions with highly enriched CLIP tags (over 100 overlapping CLIP tags in the center) were used for *de novo* consensus RNA motif analysis. Analysis of all the CLIP tags within these selected regions using the Homer algorithm revealed that GU or GGU containing sequences are statistically enriched over background (all pre-mRNA sequences) (Figure S5). This is consistent with previous reports that GGUG, GUGGU, or GGU containing RNA sequences are potential FUS binding motifs [29], [30], [40].

To validate the interaction between FUS and its own pre-mRNA, we performed anti-FUS immunoprecipitation in HeLa cells, followed by RNA purification, reverse transcription (RT) and PCR using primers specific to the introns flanking exon 7. Our results showed that indeed FUS interacted *in vivo* with its own pre-mRNAs at the region of exon 7 and its flanking introns (Figure 2C), compared to the constitutive exon 5 that was not enriched in FUS immunoprecipitates. Of note, FUS-exon7 complex (Figure 2C, lane 5) was immunoprecipitated even in the absence of UV crosslinking and under high salt wash conditions (containing 750 mM NaCl), suggesting a strong association of FUS protein with exon 7 of its own pre-mRNAs.

### The *FUS* splice variant with exon 7 skipped is subject to nonsense-mediated decay (NMD)

Skipping of *FUS* exon 7 results in an open reading frame shift and introduces a premature stop codon in exon 8. The exon 7-skipped *FUS* transcripts (NCBI RefSeq, NR_028388.2; Ensemble, ENST00000566605) are predicted to be subject to NMD. To detect the exon 7-skipped variant, we treated cells with cycloheximide (CHX) for 6 hours (h), which inhibits translation and thereby NMD [41]. *FUS* splice variants were assessed by reverse transcription (RT) and PCR using primers in exon 6 and exon 8. Indeed, we observed that exon 7-skipped splice variants were present in a variety of human and mouse cell lines including human cervical cancer cells HeLa, embryonic kidney cells HEK293, neuroblastoma cells SH-SY5Y, and mouse motor neuron cells NSC-34 (Figure 2D). Although the ratio of exon 7-skipped variants varies in different cell lines, they were all increased after CHX treatment, suggesting these variants undergo NMD. The PCR products of exon 7-skipped variants in HEK293 cells were confirmed by cloning and sequencing. At 6 h post CHX treatment, no significant changes were detected at the FUS protein level in all the cells tested (Figure S6).

### FUS is a repressor of exon 7 splicing

**Figure 3:**
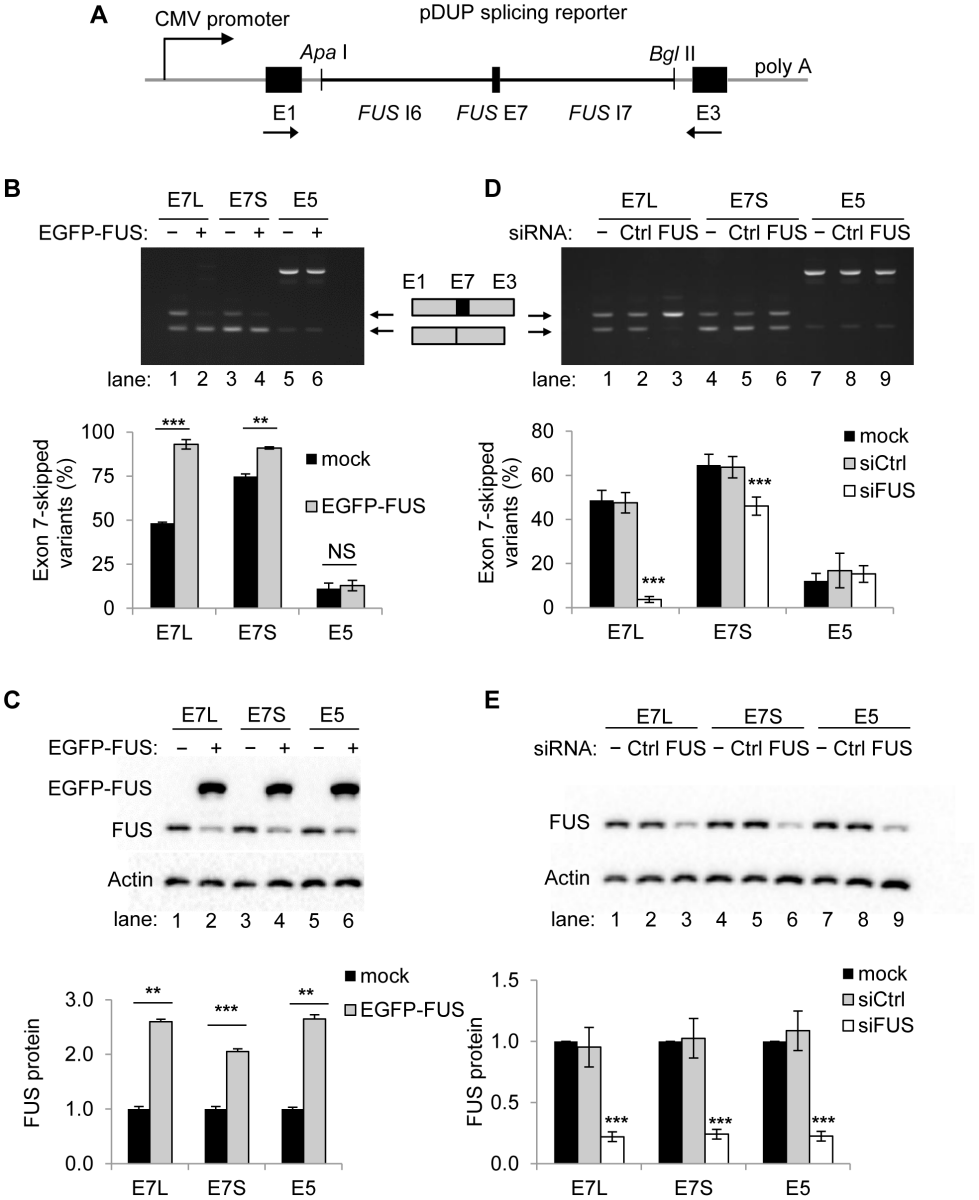
FUS is a repressor of exon 7 inclusion. A) Schematic of the pDUP-FUS-E7L, -E7S and -E5 reporters. The sequence of interest (see Methods) was inserted between the *Apa*I and *Bgl*II sites to replace the second exon in the human β-globulin pDUP splicing reporter minigene. Arrows indicate primer positions. B) RT-PCR of splice variants in pDUP-FUS-E7L, -E7S and -E5 reporters cotransfected with EGFP-FUS in HEK293 cells. The exon skipping ratio is equal to the intensity of exon-skipped band divided by the intensity sum of both splice variants. Bar graphs represent mean ± SD (n = 3). C) Western blot analysis of FUS and EGFP-FUS. β-Actin was used as a loading control. Bar graphs represent mean ± SD (n = 3). D) RT-PCR of splice variants in the pDUP-FUS-E7L, -E7S and -E5 reporters cotransfected with FUS siRNA (siFUS) or control siRNA (siCtrl) in HEK293 cells. Bar graphs represent mean ± SD (n = 3). E) Western blot analysis of FUS. β-actin was used as a loading control. Bar graphs represent mean ± SEM (n = 4). For all the quantification, student's *t*-tests were performed. ** *P*≤0.01, *** *P*≤0.001. NS indicates no significance.

We assessed the splicing of *FUS* exon 7 in the context of the splicing reporter human β globulin minigene (Figure 3A) by RT-PCR [42]. In the pDUP-FUS-E7L (Long) construct, we cloned *FUS* exon 7 and 2.8 kb of flanking introns 6 and 7 to encompass the entire conserved region enriched with FUS CLIP tags. In the pDUP-FUS-E7S (Short) construct, we cloned *FUS* exon 7 with about 300 bp of each flanking intron to assess the effects of distal intronic regions without compromising exon 7 splice sites. The pDUP-FUS-E5 construct containing *FUS* exon 5 and its flanking introns was used as a control, since exon 5 is a constitutive exon with no enrichment of FUS binding. The level of exon 7 inclusion is around 50% in the context of pDUP-FUS-E7L reporter transfected into HEK293 cells (Figure 3B, lane 1), similar to the endogenous *FUS* transcript. Interestingly, exon 7 inclusion level was lower in the pDUP-FUS-E7S reporter (Figure 3B, lane 3), suggesting additional regulatory elements in intron 6 and intron 7. This may explain why the entire region spanning intron6-exon7-intron7 is highly conserved.

To assess whether the splicing of exon 7 is affected by FUS protein levels, a gain of function assay with the EGFP-FUS plasmids and a loss of function assay with FUS siRNA were performed in HEK293 cells. Our results showed that the repression of exon 7 was enhanced significantly in both pDUP-FUS-E7L (from 48.3%±0.6% to 93.1%±2.7%, mean ± SD, n = 3) and pDUP-FUS-E7S reporters (from 74.9%±1.3% to 91.0%±0.6%) when EGFP-FUS was expressed (Figure 3B, 3C). As a control, the splicing of exon 5 in the pDUP-FUS-E5 reporter was not affected by FUS overexpression. Conversely, the level of the exon 7-skipped products decreased strikingly in the pDUP-FUS-E7L reporter (from 48.7%±4.5% to 3.7%±1.4%) when endogenous FUS protein was reduced by siRNA (Figures 3D, 3E). The exon 7-skipped splice variant in pDUP-FUS-E7S reporter was also decreased but to a lesser extent, from 64.8%±4.8% to 46.0%±4.1%, which suggests that more regulatory elements in the entire intron6-exon7-intron7 region are required to control exon 7 alternative splicing.

**Figure 4:**
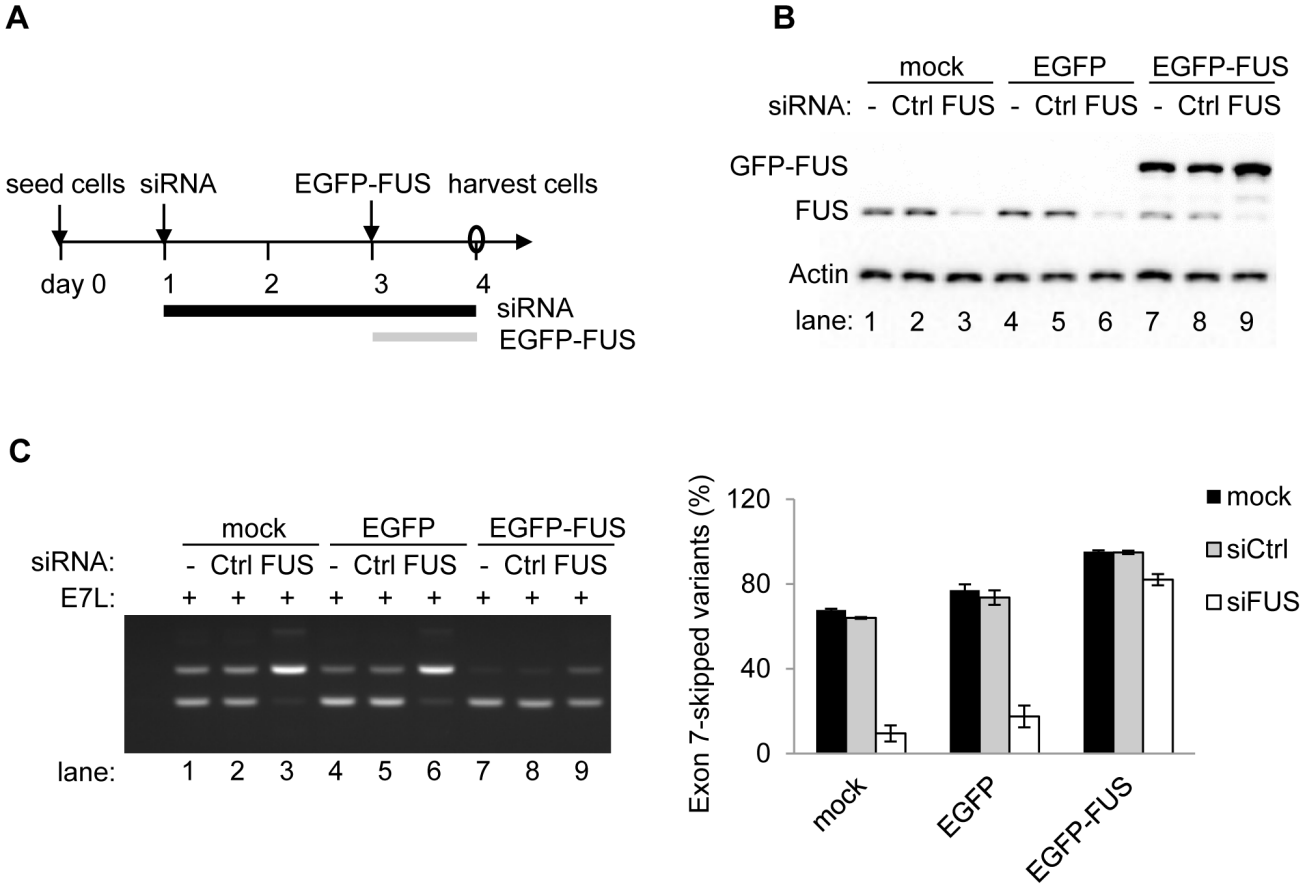
Exogenous expression of FUS can rescue the depletion of endogenous FUS to repress exon 7. A) Schematic of the rescue assay. FUS protein was first knocked down by siRNA targeting 3′ UTR of *FUS* for 48 h and then increased by the expression of EGFP-FUS plasmid for 24 h in HEK293 cells. B) Western blot analysis of FUS in the rescue assay. C) RT-PCR (left panel) of the *FUS* exon 7 splice variants of the pDUP-FUS-E7L reporter in the rescue assay. Bar graphs (right panel) represent mean ± SD (n = 3).

To further test the dependence of exon 7 splicing on FUS protein levels, a rescue assay with the pDUP-FUS-E7L reporter was performed by knocking down endogenous FUS using siRNAs that target the 3′ UTR of *FUS* pre-mRNAs, followed by expressing EGFP-FUS (Figure 4A, 4B). The level of exon 7-skipped splice variants was reduced from 67.7%±0.6% (lane 1) to 9.5%±3.8% (lane 3) at 48 h post siFUS treatment, and recovered to 82.0%±0.6% (lane 9) after introduction of EGFP-FUS for 24 h (Figure 4C). This assay strongly supports that FUS is a repressor of its own exon 7. Moreover, it also demonstrated that EGFP-FUS is as competent as the endogenous FUS to repress exon 7 splicing.

**Figure 5:**
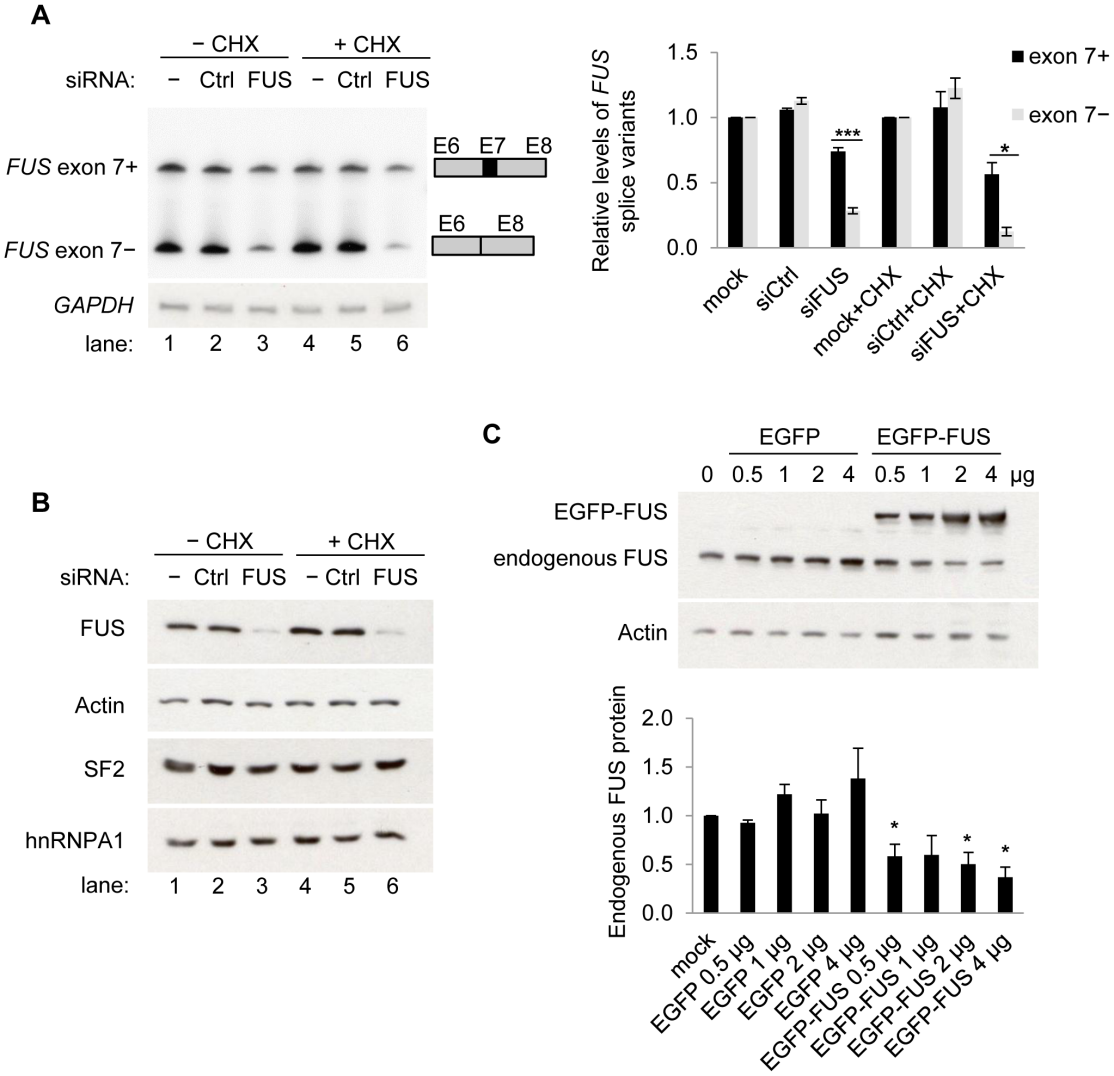
FUS represses exon 7 of the endogenous *FUS* pre-mRNA and autoregulates its own protein levels. A) FUS represses exon 7 of the endogenous *FUS* pre-mRNA. [γ-^32^P] ATP labeled RT-PCR products of endogenous *FUS* exon 7 splicing variants in HEK293 cells, following knockdown of FUS by siRNA (siFUS). Cycloheximide (CHX) was used to inhibit NMD. The reduction of each splice variant (exon 7-included or -skipped) by siRNA relative to the corresponding mock transfection was calculated (lane 2, 3 relative to lane1; lane 5, 6 relative to lane 4). GAPDH was used as a loading control. In each sample, the reduction of the exon 7-included variant was compared with the reduction of the corresponding exon 7-skipped variant using student's *t*-tests. Bar graphs represent mean ± SEM (n = 3). * *P*≤0.05, *** *P*≤0.001. B) Western blot analysis of the FUS protein and two other RNA binding proteins SF2 and hnRNPA1. Actin was used for loading control. C) Expression of EGFP-FUS downregulates endogenous FUS protein. Western blot analysis of endogenous FUS protein following expression of EGFP-FUS in HEK293 cells. Both endogenous FUS and EGFP-FUS were detected using anti-FUS antibody (10F7). β-Actin was used for loading control. The endogenous FUS protein levels were quantified. Bar graphs represent mean ± SEM (n = 3). Student's *t*-tests were performed. Samples transfected with EGFP or EGFP-FUS were compared with the control (mock transfection). * *P*≤0.05.

To determine whether FUS protein levels affect exon 7 splicing of endogenous FUS transcripts, semi-quantitative PCR using radiolabeled primers was performed to examine the *FUS* splice variants after siRNA knockdown. To prevent NMD of the exon 7-skipped *FUS* transcripts, we treated cells with cycloheximide (CHX), which allowed visualization of increases of exon 7 skipping in endogenous transcripts (Figure 5A, comparing bottom bands in lanes 4 and 5 with those in lanes 1 and 2). It is important to note that the siRNA targets both *FUS* splice variants, but the difference in the reduction of each splice variant relative to its corresponding mock transfection control is quantifiable and informative [43]. The level of exon 7-skipped variants (bottom band, lane 6) was reduced to 15% of the mock transfection (bottom band, lane 4), while the level of the exon 7-included variant (top band, lane 6) was only reduced to 50% of the mock transfection control (top band, lane 4) upon siRNA knockdown of FUS and CHX treatment (Figure 5A). A lesser reduction in exon 7-included variants than in exon 7-skipped variants was also observed without CHX treatment (lane 3). This result is consistent with the splicing reporter minigene assay, indicating that FUS is a repressor of exon 7 and reduced FUS protein levels results in less exon 7 repression. Western blot analysis confirmed siRNA knockdown of endogenous FUS protein (Figure 5B). Expression of splicing factors SF2 and hnRNPA1 were unaffected (Figure 5B), suggesting changes in *FUS* splicing were unlikely the result of an indirect mechanism.

Taken together, we have demonstrated that FUS is a key repressor of its own exon 7 in both splicing reporter assays and endogenous FUS splicing assays.

### FUS autoregulates its own protein levels

We showed that FUS repressed its exon 7 splicing and that the resultant exon 7-skipped transcripts were degraded by NMD and cannot be translated to protein. This observation led us to hypothesize that FUS can autoregulate its own protein levels by regulating the alternative splicing of exon 7. If our hypothesis is correct, we predict that exogenous expression of FUS will downregulate endogenous FUS protein levels by promoting exon 7 skipping and consequently NMD. Western blot analysis using FUS antibody detected both endogenous FUS and EGFP-FUS. The results showed that the endogenous FUS protein level was decreased by about 50% with transient expression of EGFP-FUS in HEK293 cells (Figure 5C). The endogenous *FUS* mRNA level was measured by quantitative RT-PCR (qRT-PCR) using primers annealing to the 3′ UTR of endogenous *FUS* transcripts but not the coding sequence of the EGFP-FUS transcripts. There was a slight reduction of endogenous *FUS* mRNA levels in the EGFP-FUS expressing cells, but no statistical significance was detected (Figure S7), suggesting the observed reduction in FUS protein levels occurs mainly at the post transcriptional level. Our finding of FUS autoregulation is also consistent with the observation in a *FUS* transgenic mouse model that the endogenous mouse FUS protein was reduced following overexpression of human FUS [44].

### ALS-associated FUS mutants are deficient in regulating alternative splicing and autoregulation

The majority of ALS-associated *FUS* mutations occur within the region coding for the nuclear localization signal, resulting in cytoplasmic retention of FUS [5] and inferred loss of FUS function in the nucleus. We propose that FUS autoregulation is deficient in ALS mutants due to the alteration of their cellular localization, which results in compromised FUS-dependent splicing regulation. To test this hypothesis, we made EGFP-FUS constructs with the ALS-associated mutations R521G, R522G and ΔE15 (deletion of last 12 amino acids in the C-terminus), which respectively correlates with minor, moderate, and severe cytoplasmic accumulation of FUS, as reported by others both in ALS patients and in cell culture systems [6], [8], [9], [11]. As a control, an RNA binding incompetent mutant EGFP-FUS RRM 4F-L was made by mutating four phenylalanine (F) to leucine (L) (F305L, F341L, F359L, and F368L) in the RNA recognition motif (RRM) [45]. We tested the effects of these mutants on FUS cellular localization, exon 7 splicing and autoregulation in HEK293 cells.

**Figure 6:**
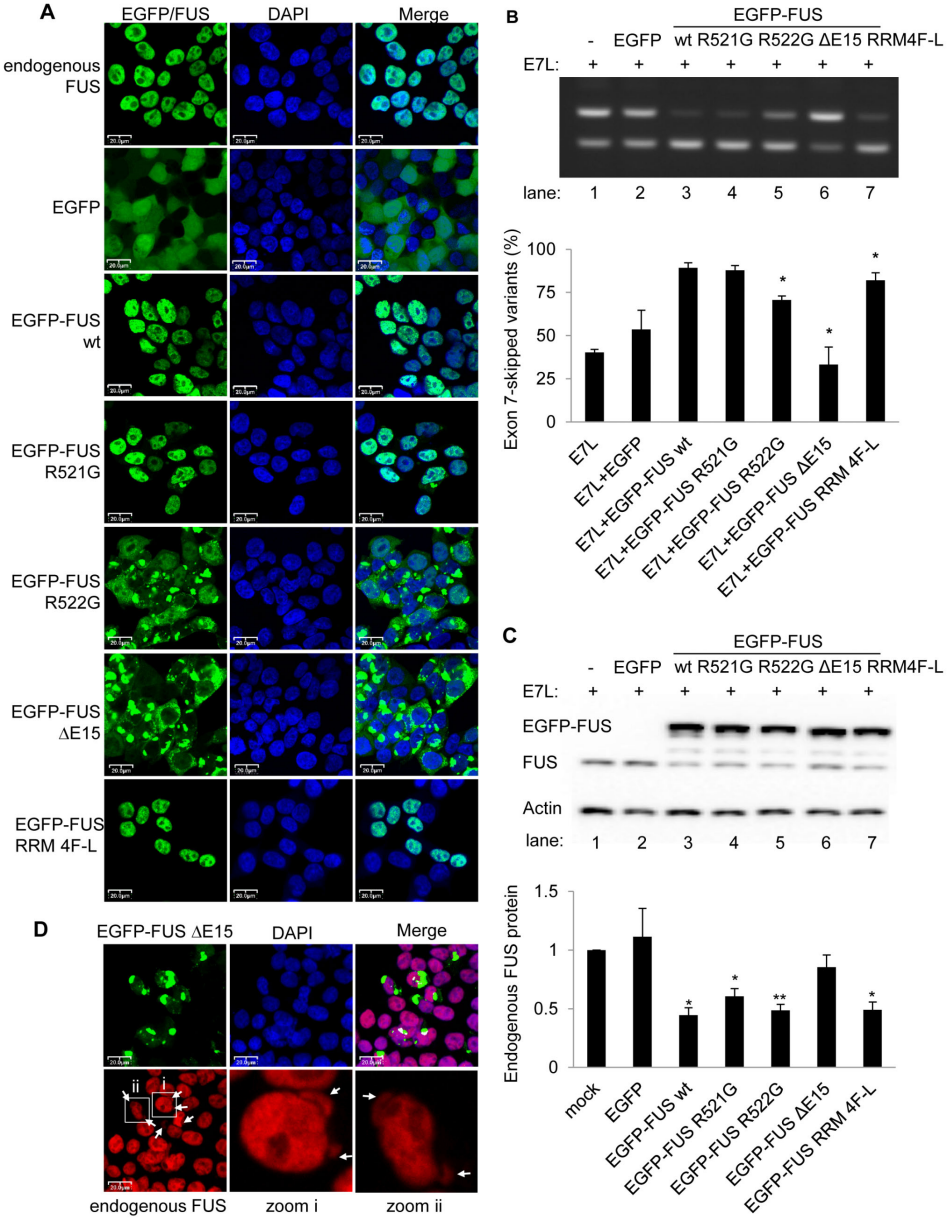
ALS-associated FUS mutants are deficient in alternative splicing and autoregulation. A) Confocal fluorescent microscopy showing the cellular localization of EGFP-FUS mutants in HEK293 cells. Magnification, 40×. Scale bar, 20 µm. Endogenous FUS protein was detected using anti-FUS antibody (Bethyl, BL1355). DNA in the nucleus was stained with DAPI or NucRed Dead 647. B) RT-PCR analysis of *FUS* exon 7 splice variants in pDUP-FUS-E7L coexpressed with either wildtype (wt) or mutant EGFP-FUS in HEK293 cells. Bar graphs represent mean ± SD (n = 3). EGFP-FUS mutants were compared with EGFP-FUS wildtype protein using student's *t*-tests. C) Western blot analysis of endogenous FUS and EGFP-FUS protein in HEK293 cells, using anti-FUS antibody (10F7). Bar graphs represent mean ± SEM (n = 3). EGFP, EGFP-FUS wildtype and mutants were compared with mock transfected cells using student's *t*-tests. In both panel B and C, * *P*≤0.05, ** *P*≤0.01. D) Confocal fluorescent microscopy showing the localization of endogenous FUS protein in the cytoplasmic aggregates of EGFP-FUS ΔE15 mutants expressed in HEK293 cells. Anti-FUS antibody (Bethyl, BL1355) recognizing C-terminus epitope detected only endogenous FUS protein but not ΔE15 mutants. Magnification, 40×. Scale bar, 20 µm.

Consistent with previous reports [6], [8], [9], [11], we observed predominant nuclear localization of wildtype FUS, minor cytoplasmic and mainly nuclear localization of R521G, moderate cytoplasmic accumulation and aggregation of R522G, and severe cytoplasmic aggregation with much less nuclear localization of the FUS ΔE15 mutant, following transient transfection in HEK293 cells (Figure 6A) and mouse motor neuron cells NSC-34 (Figure S8). The RRM mutant, like the wildtype FUS protein, was predominantly localized in the nucleus.

To test the function of the FUS mutants in exon 7 alternative splicing, the splicing reporter minigene pDUP-FUS-E7L together with either wildtype or mutant EGFP-FUS plasmids were transfected into HEK293 cells (Figure 6B). Expression of wildtype EGFP-FUS protein resulted in repression of exon 7 as expected, with an increase of the exon7-skipped products from 40.4%±1.7% to 89.3%±3.0% (mean ± SD, n = 3). Expression of the ALS-associated mutants, compared to the wildtype FUS, resulted in significantly compromised repression of exon 7 (*P*≤0.05, n = 3). The more cytoplasmic localization of the ALS mutants, the less exon 7 repression, with exon 7 skipping ratio of 87.6%±2.8% for R521G, 70.6%±2.4% for R522G and 33.3%±10.1% for ΔE15 mutant. Expression of the EGFP-FUS RRM 4F-L mutant only resulted in a mild reduction of exon 7 repression with a ratio of exon 7 skipping of 82.1%±4.3%, which was statistically different from the EGFP-FUS wildtype protein (*P*≤0.05, n = 3). While the RRM 4F-L mutant was reported incompetent to bind RNA [45], its expression did increase the nuclear concentration of FUS protein, which may result in more endogenous FUS binding the intron6-exon7-intron7 region in the reporter minigene and repression of the exon 7 splicing. This may explain the slight reduction of exon 7 repression when the RRM 4F-L mutant was expressed. Our data suggest that both the nuclear concentration of FUS protein and RNA binding are critical for the regulation of *FUS* exon 7.

Consistent with the splicing data, western blot analysis confirmed that increased exon 7 skipping led to a decrease in endogenous FUS protein levels by 55.5%, when EGFP-FUS wildtype protein was expressed (Figure 6C). Conversely expression of various EGFP-FUS ALS mutants, which showed reduced nuclear localization and exon 7 skipping, resulted in less reduction of the endogenous FUS protein (Figure 6C). Expression of the ΔE15 mutant that is predominantly localized in the cytoplasm only downregulated the endogenous FUS protein by 14.6%. This mild downregulation of FUS protein is consistent with little increase of exon 7 skipping observed in the splicing assay (Figure 6B). We also confirmed in human neuroblastoma cells SH-SY5Y that ALS-associated FUS mutants were deficient in regulating exon 7 repression in the context of the pDUP splicing reporter minigene, and that the deficiency correlated with the extent of cytoplasmic localization of the mutants (Figure S9).

We observed that wildtype endogenous FUS protein was co-localized with the cytoplasmic aggregates of FUS ΔE15 mutant in both HEK293 cells (Figure 6D) and mouse motor neuron cells NSC-34 (Figure S8), using an antibody which detects only the endogenous FUS protein but not the ΔE15 mutant by recognizing a C-terminal FUS epitope. These data suggest that the FUS mutants may sequester the wildtype FUS protein in the cytoplasm and further contribute to the aggregation. This is consistent with a recent report that GFP-FUS ALS mutant is co-localized with MYC-FUS wildtype protein in the cytoplasmic aggregates [46]. We report here the localization of the endogenous FUS protein in the cytoplasmic aggregates of ALS-associated FUS mutants.

Taken together, our data suggest that the severity of FUS cytoplasmic accumulation correlates with the deficiency of exon 7 repression and autoregulation of FUS protein levels. The deficiency of ALS-associated FUS mutants in alternative splicing and autoregulation may exacerbate the cytoplasmic accumulation of ALS-associated FUS mutants.

### Exon 7 skipping can be induced by antisense oligonucleotides

**Figure 7:**
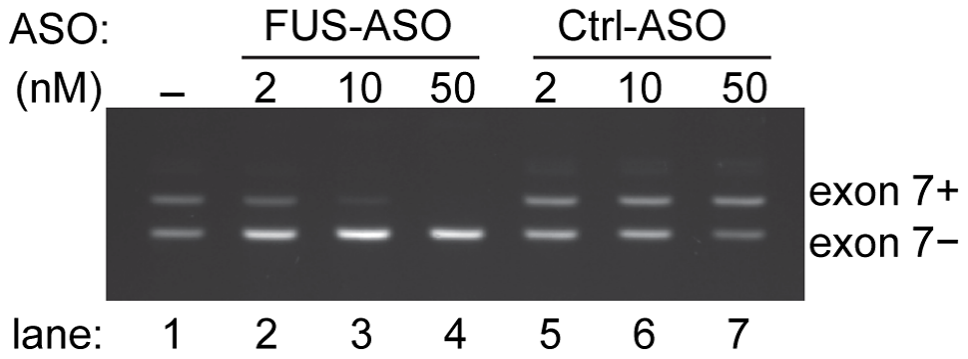
Modulation of *FUS* exon 7 alternative splicing by antisense oligonucleotides. An antisense oligonucleotide targets the splice junction of *FUS* intron 6 and exon 7 (FUS-ASO). The ASO targeting *SRA* was used as control (Ctrl-ASO). The pDUP-FUS-E7L reporter was cotransfected with increasing amount of FUS-ASO or Ctrl-ASO. Splice variants with *FUS* exon 7-included or -skipped were assessed by RT-PCR and separated on an agarose gel containing ethidium bromide.

Our data showed FUS autoregulation is deficient in cells expressing ALS-associated FUS mutants. Moreover, this deficiency increases with the relative severity of the cytoplasmic accumulation of individual FUS mutants, which would be expected to exacerbate the rate of cytoplasmic accumulation and FUS proteinopathy. Use of splicing-modulating antisense oligonucleotides (ASOs) is a therapeutic strategy of great potential to treat diseases arising from splicing defects [47]–[49]. We rationalized that ASOs promoting *FUS* exon 7 skipping should mimic the repression of exon 7 by FUS and thereby have the potential to restore the deficient FUS autoregulation in patients with ALS-associated *FUS* mutations. We designed FUS-ASOs to target the junction of intron 6 and exon 7. FUS-ASOs were synthesized using 2′-O-methyl-oligoribonucleotides with phosphorothioate linkages to increase ASO stability and then tested with the pDUP-FUS-E7L minigene in HEK293 cells. Our results showed that FUS-ASOs induced repression of exon 7 in a dose dependent manner (Figure 7). This suggests a possibility that deficient FUS autoregulation can be therapeutically restored to reduce or alleviate the extent of abnormal FUS cytoplasmic accumulation occurring in ALS patients with *FUS* mutations.

## Discussion

**Figure 8:**
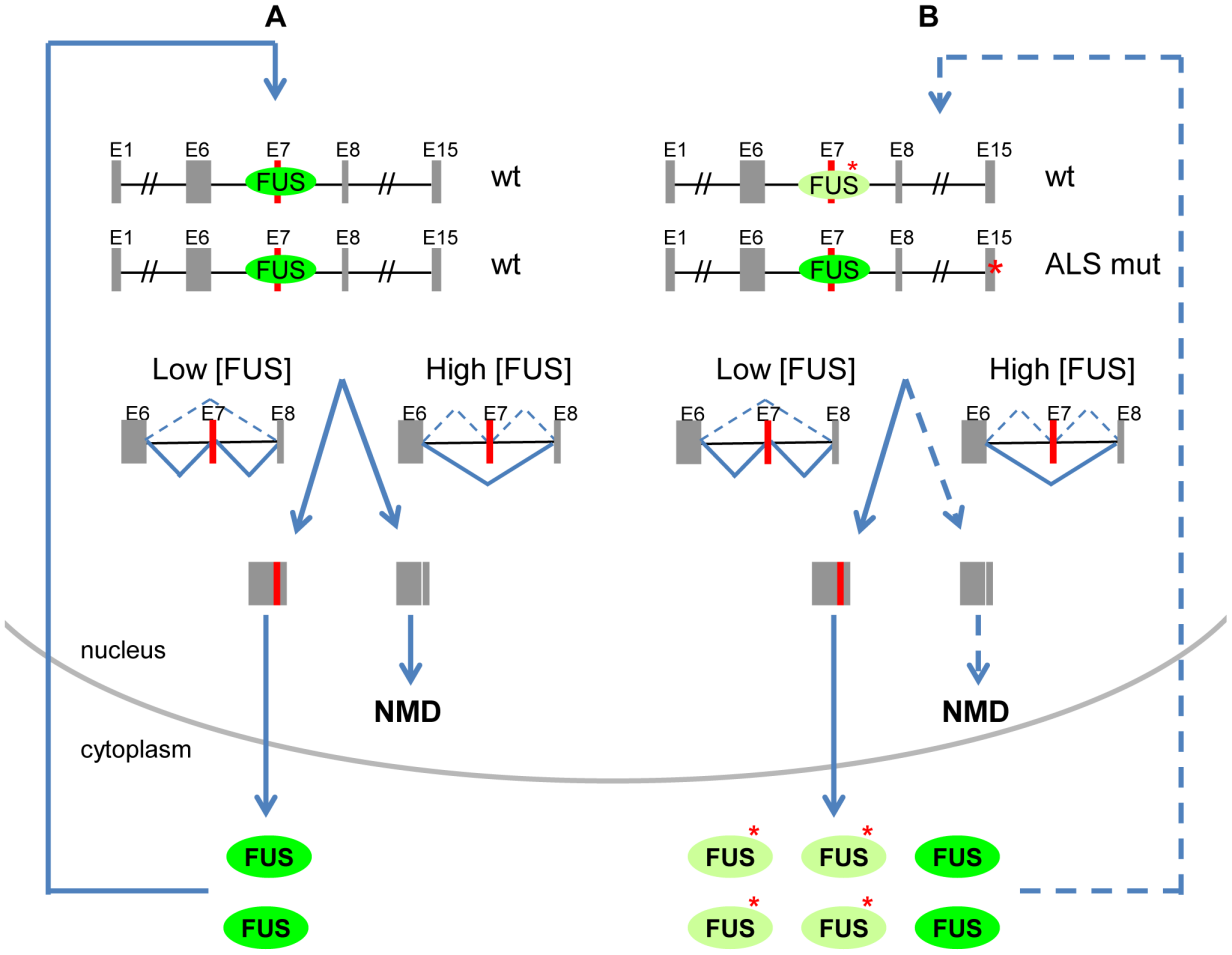
Proposed model of FUS autoregulation and its contribution to ALS pathogenesis. A) A model illustrates FUS autoregulation as a feedback loop to control the homeostasis of FUS protein levels. When FUS protein levels are high, FUS downregulates its own protein by repressing exon 7 inclusion to produce exon 7-skipped transcripts for NMD. When FUS protein levels are low, the repression of exon 7 is reduced and more splice variants with exon 7-included are produced for translation. B) The deficient FUS autoregulation in ALS-associated FUS mutants may contribute to its abnormal cytoplasmic accumulation. Mutations within the nuclear localization signal result in cytoplasmic retention of FUS mutants and reduction of nuclear FUS. The reduction of nuclear FUS leads to the reduction of *FUS* exon 7 repression, which would in turn produce more exon 7-included transcripts for translation, thereby driving elevated FUS protein synthesis. This compromised FUS autoregulation forms a feed-forward loop, potentially exacerbating the abnormal cytoplasmic accumulation of FUS mutants. *FUS* mutations and FUS mutants are indicated with a red asterisk.

Here we report a novel autoregulatory mechanism of FUS by alternative splicing and NMD. The model shown in Figure 8A illustrates FUS autoregulation as a feedback loop to control the homeostasis of FUS protein levels. High levels of FUS protein lead to increased FUS binding to exon 7 and its flanking introns, promoting exon 7 skipping and NMD to reduce excessive FUS protein. Low levels of FUS protein would favor exon 7 inclusion, resulting in increased FUS protein production. Alternative splicing-mediated NMD and highly conserved intronic sequences represent an emerging common mechanism utilized by RNA binding proteins (RBPs) to maintain their homeostasis [43], [50], [51]. FUS now joins this increasing list of autoregulated RBPs, including PTB, hnRNP L, Nova and TDP-43 [43], [50]–[52]. FUS regulates many aspects of gene expression including transcription, alternative splicing and RNA transportation [23]–[25]. Dynamic regulation and conserved targets suggest it is important to keep these functional activities of FUS in tight control, and that FUS likely has a co-factor role in coordinating them. For example, loss of *FUS* can cause genomic instability and developmental defects in mouse, *Drosophila* and Zebrafish [18], [19], [53]. Conversely, high levels of FUS are associated with cancer and ALS, and moreover, are known genetic determinants of these diseases. Overexpression of *FUS* is observed in liposarcoma and leukemia with *FUS* translocations [54], [55]. Aberrant accumulation of FUS mutant protein is a characteristic pathology of FUS-associated ALS [8], [9]. Depletion of FUS in the mouse nervous system affects the abundance or the splicing of about 1000 mRNAs [30], suggesting that maintaining equilibrated FUS protein levels is critical for RNA processing.

In ALS, another frequently mutated gene TDP-43 is also an RNA binding protein that autoregulates its own protein levels [52], [56]. The direct mechanism of TDP-43 autoregulation is different from what we report here for FUS. TDP-43 binds to the 3′ UTR of its own pre-mRNA to trigger either NMD [56] or exosome-dependent degradation [52]. To our knowledge, we are the first to report a FUS autoregulatory mechanism through alternative splicing and NMD. Autoregulation of both FUS and TDP-43 by post transcriptional mechanisms suggests their functional activities are tightly controlled and that unbalancing of this regulation may underpin molecular mechanisms that promote neurodegeneration in ALS. Mice and rats expressing TDP-43 without the autoregulatory sequence developed more severe neurodegeneration than those expressing autoregulated wildtype or ALS-linked TDP-43 mutants, strongly suggesting deficient TDP-43 autoregulation contributes to neurodegeneration [57]. This is also likely the case for FUS autoregulation, which needs to be experimentally tested in rodent models. Interestingly, in both FUS-associated ALS and cancer, loss of heterozygosity of the *FUS* gene is never observed. This suggests, at least genetically, that while compromised FUS autoregulation contributes to the initiating or driving events resulting from *FUS* mutations in these diseases, the activity of the wild-type *FUS* allele is required or selected to maintain this pathological state. In this regard, it is important to understand how both mutant and wild-type FUS activities may contribute to the progression of ALS and cancer.

Compromised FUS homeostasis by autoregulation is expected in cells harbouring ALS-associated mutations (Figure 8B). The majority of ALS-associated mutations are located in the nuclear-localization signal (NLS) of *FUS*, resulting in both a cytoplasmic retention of FUS mutants and a reduction of FUS protein levels in the nucleus. The reduction of nuclear-localized FUS leads to the reduction of *FUS* exon 7 repression, which in turn likely induces production of more exon 7-included transcripts for translation, thereby driving elevated protein synthesis of FUS. In our model of FUS autoregulation, a deficiency of FUS in the nucleus would favour a feed-forward mechanism, and thereby actually exacerbate the abnormal cytoplasmic accumulation of FUS mutants. Our observation that endogenous wildtype FUS protein was co-localized with the cytoplasmic aggregates of FUS mutants in both HEK293 cells and NSC-34 motor neuron cells suggests that the FUS mutants may further sequester the wildtype FUS protein in the cytoplasm and form more aggregates. In ALS, FUS cytoplasmic accumulation is a progressive process and increases with disease duration [58]. The deficient FUS autoregulation may lead to long term detrimental effects, and could be part of the mechanism underlying age-dependent neurodegeneration and death of neurons with ALS-associated *FUS* mutants.

Indeed, a genotype-phenotype relation between different *FUS* mutations and FUS cytoplasmic accumulation or the age of ALS onset is observed [8], [11]–[13]. The stronger the NLS mutation (severity of cytoplasmic retention), the earlier the age of ALS onset. The three *FUS* mutants we constructed, R521G, R522G and ΔE15 (last 12 amino acids truncation) represent minor, moderate and severe cytoplasmic accumulation, respectively. The reported mean age of disease onset is 43 for R521G, 28.5 for R522G and 18 for R495X (last 32 amino acid truncation) in the later generation [8], [11]. Here, we demonstrated that in HEK293 cells and SH-SY5Y cells expressing these same R521G, R522G and ΔE15 *FUS* mutants, increased cytoplasmic localization of FUS directly correlated with increased deficiencies of exon 7 skipping and FUS autoregulation. We speculate that *FUS* exon 7-skipped splice variants are reduced in the tissues or cell lines derived from ALS patients with *FUS* mutations, which can be further experimentally tested.

Regulated splicing of exon 7 is a good model to examine FUS-dependent alternative splicing in detail, since it is one of the most significant FUS CLIP clusters identified in our FUS CLIP-seq. Our finding is also consistent with the report that FUS binds to highly conserved introns of genes encoding RNA binding proteins [32]. *FUS* exon 7 and its flanking introns were also identified previously as RNA targets of FUS and of TDP-43 respectively from FUS CLIP-seq and TDP-43 CLIP-seq of mouse brains [30], [56], but the functional significance of this implicated region was not experimentally tested. Interestingly, the prime molecular target of two genetic determinants of ALS converges on the same highly conserved *FUS* alternative exon and its flanking introns. This makes a compelling argument that the processing of *FUS* pre-mRNA specifically, and by extension, the role of FUS in alternative exon splicing in general, is an important molecular determinant of ALS. Lagier-Tourenne *et al*. proposed that FUS binding to *FUS* intron6-exon7-intron7 may result in retention of intron 7 to make a shorter *FUS* transcript with an alternative 3′ UTR [30]. We noticed that both NCBI RefSeq database (NR_028388.2) and Ensemble database (ENST00000566605) annotated a *FUS* splice variant without exon 7, which is predicted to undergo NMD. We experimentally validated that the exon 7-skipped variant of FUS was expressed in multiple human and mouse cell lines and that the steady state levels of the exon 7-skipped variant was increased after inhibition of NMD. Furthermore, we demonstrated that FUS is a repressor of its own exon 7 by splicing assays of both splicing reporter minigenes and endogenous *FUS* pre-mRNAs.

FUS-regulated alternative splicing of cassette exons is not just limited to its own exon 7. We found FUS CLIP clusters were significantly associated with alternative splicing events of cassette exons. Our normalized complexity map of 87 FUS-associated cassette exon events revealed that FUS CLIP clusters were enriched in the introns flanking cassette exons, proximal to upstream 3′ splice sites and downstream 5′ splice sites, with the highest peak overlapping the downstream 5′ splice sites. FUS binding proximal to 5′ splice sites suggests that FUS may be associated with the assembly of spliceosome at 5′ splice sites, consistent with previous reports that FUS, as well as the related family member TAF15, are in the U1 snRNP (small nuclear ribonucleoprotein) complex [59], [60]. FUS binding proximal to 3′ splice sites suggests it may also affect the spliceosome assembly at 3′ splice sites; this functional significance is yet to be determined [31]. Activation or repression of cassette exon splicing can be dependent on RNA binding positions, as suggested in Nova [36] and FOX2 [61] CLIP-seq data, which showed that Nova and FOX2 binding proximal to 3′ splice sites repressed cassette exons, while conversely binding proximal to 5′ splice sites promoted cassette exons. However, splicing factors such as hnRNP A1 and PSF binding to introns proximal to 5′ splice sites can also repress cassette exons [62], [63]; which suggests that activation or repression of cassette exon splicing is likely more complex. We experimentally demonstrated in this paper that FUS is a repressor of its own exon 7. However, it does not rule out the possibility that FUS may activate other cassette exons. The repression or activation of a given cassette exon by FUS in a tissue specific manner might be controlled by different signaling pathways and/or cell type specific splicing factors involved in the complex in a spatio-temporal manner.

Sequence motif analysis of FUS CLIP clusters in our data set did not identify a significant common consensus motif (data not shown). We found variable motifs throughout the four binding peaks in the complexity map (data not shown), suggesting limited or context-dependent FUS binding specificity, consistent with previous reports [28], [29], [31], [32]. Analysis of CLIP clusters within the region encompassed by the highest binding peak in the normalized complexity map (5′ splice site downstream of cassette exons) did identify an enrichment of a CAGGUU motif, which is similar to the human 5′ splice site consensus sequence MAG|GURAGU [38]. While this might be expected, it is interesting to note that GGU is the most common FUS binding site consensus sequence. This was originally reported in a SELEX assay (GGUG) [40], and subsequently by CLIP-seq analysis from Lagier-Tourenne *et al*. (GUGGU) [30] and Rogelj *et al.* (GGU) [29]. A detailed examination of consensus RNA motifs within *FUS* intron 6 and intron 7 did reveal that GU or GGU containing sequences were statistically enriched, which provides FUS intron6-exon7-intron7 as a model for further experimental determination of FUS binding sites.

FUS CLIP-seq data and RNA-seq data revealed a wide range of pre-mRNAs as candidate targets of FUS [28]–[32], [64]. However, the biological significance of FUS-regulated RNA processing is only now being examined. Here we demonstrated that FUS-regulated cassette exon splicing of its own pre-mRNA leads to NMD, suggesting that FUS-regulated alternative splicing may be a common post transcriptional mechanism for the regulation of gene expression. This function of FUS in regulating cassette exon splicing is likely conserved in different tissues and species, since FUS-associated cassette exons were also observed in human and mouse neural tissues [28]–[30]. Our evidence demonstrating FUS is a splicing repressor of its exon 7 strongly supports the hypothesis that alternative splicing of many other neuronal- and disease-associated genes containing FUS-targeted cassette exons may also be regulated by FUS.

In conclusion, our study uncovers an autoregulatory mechanism of *FUS* expression through alternative splicing and NMD, and demonstrates that its function in splicing regulation is deficient in ALS-associated FUS mutants. This study addresses a biological significance of FUS-regulated alternative splicing, and its potential relevance to ALS pathogenesis. Furthermore, our findings have important implications for the development of new therapeutic approaches to target alternative splicing in treating ALS. Taking advantage of our findings, splicing-modulating antisense oligonucleotides can be developed to induce exon 7 skipping and produce the *FUS* splice variants undergoing NMD. This may be a promising strategy to reduce the abnormal FUS cytoplasmic accumulation in ALS. Moreover, FUS autoregulation by alternative splicing provides insight into a molecular mechanism by which FUS-regulated pre-mRNA processing can impact a significant number of targets important to neurodegeneration.

## Materials and Methods

### Cell culture

Human cervical cancer cell line HeLa (ATCC, CCL-2) and human embryonic kidney cell line HEK293 (ATCC, CRL-1573) were grown in DMEM with 10% bovine growth serum (HyClone, SH30541.03). Human neuroblastoma cell line SH-SY5Y (gift from Dr. Louise Simard) was cultured in MEM-F12 medium (1∶1) supplemented with 10% fetal bovine serum (GIBCO, 12483). Mouse motor neuron cell line NSC-34 (gift from Dr. Louise Simard) was cultured in DMEM with 10% fetal bovine serum.

### Cross-linking immunoprecipitation and sequencing (CLIP-seq)

CLIP-seq was performed as described [65]. Briefly, HeLa cells on 15 cm dishes were UV crosslinked *in vivo* at 400 mJ/cm^2^ using Stratalinker (Stratagene 1800). Cell lysates were prepared in lysis buffer [65], followed with partial RNase A (Sigma, R6513) digestion at different final concentrations ranging from 0.001 µg/ml to 0.1 µg/ml. FUS-RNA complexes were immunoprecipitated using mouse monoclonal anti-FUS antibody 10F7 pre-bound with protein G agarose beads (Pierce, 22851). 10F7 is a mouse monoclonal antibody previously developed in the Hicks laboratory by immunizing BALB/c mice with GST (glutathione-s-transferase)-FUS fusion protein. Various clones were screened using western blotting, immunocytochemistry, and flow cytometry, and the 10F7 antibody performed best for all three methods. This antibody recognizes amino acids 34–51 of FUS. Immunoprecipitation using mouse IgG (Sigma, I5381) prebound with protein G agarose beads was performed in parallel as a control. While FUS-RNA complexes were still bound to beads, the 5′ end of CLIP RNA was radiolabeled with [γ-^32^P] ATP, and the 3′ end of CLIP RNA was ligated to a 3′ RNA linker. The radiolabeled FUS-RNA complex was separated onto a 10% (w/v) Bis-Tris gel (Novex NuPAGE), transferred to a nitrocellulose membrane (Bio-Rad, 162-0115), and exposed to X-ray film (Amersham Hyperfilm MP) for autoradiography. The appropriate shifted FUS-RNA bands were cut out of the nitrocellulose membrane and subject to protein digestion using proteinase K (Roche, 3115879001). RNA was recovered using phenol chloroform extraction and sodium acetate, ethanol-isopropanol (1∶1) precipitation. The recovered RNA was further ligated to a 5′ RNA linker, and subject to DNase (Promega, M6101) digestion and RNA recovery again. The final RNA product was reverse transcribed to cDNA using linker specific primers and subject to deep sequencing using the Illumina Genome Analyzer II (single end, 72 bp) at the Center of Applied Genomics in Toronto (TCAG).

### Bioinformatics

Sequences of unique CLIP tags were mapped to the human genome (GRCh37) by BlastN [66] after trimming the CLIP linker sequences and removing duplicate CLIP tags. A peak finder algorithm CisGenome (www.biostat.jhsph.edu/~hji/cisgenome/) [33] was used to define CLIP clusters with significant enrichment (FDR≤0.05; FUS CLIP *vs* control mouse IgG CLIP). The RNA targets from our FUS CLIP-seq data in HeLa were compared to the RNA targets previously identified by other FUS CLIP-seq, PAR-CLIP and RIP-ChIP in different tissues and cells [28]–[32], [34]. If the gene list was not reported in the paper, the raw data of deposited fastq files [28]–[30] were retrieved from ENA (The European Nucleotide Archive, http://www.ebi.ac.uk/ena/) and uploaded to Galaxy (http://main.g2.bx.psu.edu/) [67] for sequence analysis. The CLIP-seq reads were mapped to mouse genome (mm9) using Bowtie (version 1.1.2) [68] built in Galaxy, with parameters reported in the corresponding paper. The same peak finder algorithm CisGenome was used to identify the CLIP clusters in all the datasets. Mouse genes were converted to the HGNC-approved human gene symbols (http://www.genenames.org/) to facilitate the comparison of different datasets. To identify the overlapping and the non-overlapping genes between different datasets, lists of the genes from the datasets were loaded into Venn, a web-based Venn diagram program, or Venn Diagram Plotter, a PC-based Venn diagram program (http://omics.pnl.gov/).

To identify our FUS CLIP clusters overlapping alternative splicing events, the genomic coordinates of CLIP clusters were searched against the UCSC Known AltEvent database (hg 19) [69] as described previously [35], [70]. Out of all 206 FUS-associated cassette exons, 87 cassette exons which are flanked by constitutive exons were used to generate a normalized complexity map as previously described [36]. FUS CLIP tags within 500 nucleotides upstream and/or downstream of these cassette exons and flanking exons were mapped. Control was an average of 100 sets of normalized complexity of 87 constitutive exons randomly selected from genes expressed in HeLa cells as determined by RNA-seq [71].

Analysis of *de novo* consensus RNA motif enrichment was performed using findMotifsGenome perl script of the Homer software [37] with parameters of 5 or 6 bases for motif length, 42 for target size, and –RNA option. Gene Ontology (GO) analysis was performed using DAVID Bioinformatics Resources 6.7 (http://david.abcc.ncifcrf.gov/) [39] or Enrichr (http://amp.pharm.mssm.edu/Enrichr/index.html) [72].

### RNA immunoprecipitation (RNA-IP)

Immunoprecipitation of FUS protein was performed using mouse monoclonal anti-FUS antibody (10F7). FUS-bound RNA was recovered using phenol-chloroform extraction and sodium acetate, ethanol–isopropanol (1∶1) precipitation, as described previously [65]. DNA was removed by DNase treatment (Ambion, AM1906). The recovered RNA was reverse transcribed to cDNA using Superscript III (Invitrogen) and amplified using Phusion Hot Start Polymerase (NEB). Primers used for amplifying the region flanking *FUS* exon 7: 5′-ACAACCTTTTGTAGCCGTTGGAAG-3′ (forward), 5′-CTTTCTGGAGGTGGTTCTGGACAC-3′ (reverse). Primers used for amplifying the region flanking *FUS* exon 5: 5′-TCCCTAGTTACGGTAGCAGTTCTC-3′ (forward), 5′-GCTGCAGACAAAGCTGAAGACATC-3′ (reverse). PCR products were resolved on a 2% agarose gel and visualized by ethidium bromide staining.

### Radiolabeled PCR of *FUS* splicing variants

Cycloheximide (CHX; Sigma) was added to cell culture medium at the final concentration of 100 µg/ml to inhibit translation and thereby nonsense mediated decay (NMD). At 6 h post CHX treatment, cytoplasmic RNA was extracted using RNeasy kit (QIAGEN) as per manufacture's recommendation. Reverse transcription (RT) was performed using Superscript III reverse transcriptase (Invitrogen). Radiolabeled PCR was performed to amplify *FUS* exon 7 splice using Phusion Hot Start DNA Polymerase (NEB). Primers were designed to anneal to exon 6 and exon 8. Primers: 5′-AGTGGTGGCTATGAACCCAGAGGT-3′ (forward), 5′-AGTCATGACGTGATCCTTGGTCCC-3′ (reverse). The reverse primer was labeled with [γ-^32^P] ATP using T4 PNK (NEB). PCR products were resolved on a 6% polyacrylamide/8M urea denaturing gel. The gel was dried, exposed to a phosphorimager plate (Kodak). The images of radioactivity signals were captured by a phosphorimager (Bio-Rad, Personal FX). The density of the radioactive bands was quantified using the Image J program v1.44p (NIH, Bethesda, MD, USA, http://rsbweb.nih.gov/ij/).

### Plasmid construction and mutagenesis assay

*FUS* cDNA was amplified from human fetal liver pAct2 cDNA library (Clontech). EGFP-FUS expression construct was made by subcloning the open reading frame of *FUS* cDNA (RefSeq: NM_004960.3) into the *Bgl*II and *Kpn*I sites of pEGFP-C1 plasmid (Clone Tech). Mutagenesis of ALS-associated mutations (R521G, R522G) and deletion (ΔE15) were performed using Quickchange Lighting Site-directed Mutagenesis Kit as per manufacturer's recommendation (Stratagene). The EGFP-FUS RNA recognition motif (RRM) mutant 4F-L (F305L, F341L, F359L, and F368L) was generated using the QuikChange Lightning Multi-Site-Directed Mutagenesis kit as per manufacturer's recommendation (Stratagene). EGFP-FUS and mutant constructs were verified by DNA sequencing.

### Immunofluorescence imaging

EGFP or EGFP-FUS (wildtype or mutant) plasmids were transiently transfected into HEK293 cells, or NSC-34 cells using Lipofectamine 2000 (Invitrogen) reagent as per manufacturer's recommendation. Cells were fixed with 4% paraformaldehyde. To detect the endogenous FUS protein, cells were incubated with the primary antibody rabbit anti-FUS (Bethyl Laboratories, BL1355) and the secondary antibody Alexa Fluor 568 or Alexa Fluor 488 donkey anti-rabbit antibody (Invitrogen). Nuclei were counter stained with DAPI or NucRed Dead 647 dye (Invitrogen). The localization of EGFP-FUS, mutants, and endogenous FUS protein was imaged using an Olympus FV500 confocal microscope and analyzed with Fluoview software version 4.3 (Olympus). Line sequential scanning was applied to avoid the potential bleed-through of fluorescence.

### Splicing reporter minigene assay

Splicing reporter minigene pDUP-FUS constructs: *FUS* exon 7 and its flanking introns were amplified using Phusion Hot Start Flex Polymerase (NEB) from human genomic DNA extracted from HEK293 cells. The sequence of interest was subcloned between the *Apa*I and *Bgl*II sites of the splicing reporter minigene pDUP175 plasmid [42]. Three reporters were made. The pDUP-FUS-E7L (Long) construct contains *FUS* exon 7, and 1453 bp upstream and 1355 bp downstream of the flanking introns. The pDUP-FUS-E7S (Short) construct contains *FUS* exon 7, 292 bp upstream and 321 bp downstream of the flanking introns. The pDUP-FUS-E5 construct is a control construct containing the sequence of *FUS* exon 5 and its flanking introns.

Splicing reporter minigene assay with FUS overexpression: 0.5 µg of pDUP reporter and 2 µg of EGFP-FUS plasmid or EGFP-FUS mutants were transfected into HEK293 cells using Lipofectamine 2000 (Invitrogen) reagent as per manufacturer's recommendation. At 48 h post transfection, cytoplasmic RNA was purified using RNeasy kit (QIAGEN). RT-PCR was performed to assess the splicing of *FUS* exon 7 using Superscript III reverse transcriptase (Invitrogen) and Phusion Hot Start DNA Polymerase (NEB). Primer sequences: 5′-CTCAAACAGACACCATGCATGG-3′ (forward) and 5′-CAAAGGACTCAAAGAACCTCTG-3′ (reverse). PCR products were resolved on a 3% agarose gel with ethidium bromide staining, and imaged using Fusion FX imager (Vilber Lourmat). The intensity of PCR bands was quantified using ImageJ software as described above.

Splicing reporter minigene assay with siRNA knockdown of FUS: 0.5 µg of the pDUP-FUS-E7L reporter and 20 nM (final concentration) of FUS siRNA (Dharmacon ON-TARGETplus SMARTpool) were transfected into HEK293 cells using Lipofectamine 2000 reagent (Invitrogen) as per manufacturer's recommendation. At 48 h post transfection, cytoplasmic RNA was purified; and splice variants were analyzed by RT-PCR and gel electrophoresis, as described above.

Rescue assay: Endogenous FUS protein was knocked down by transfecting HEK293 cells with a custom designed siRNA targeting 3′ UTR of human FUS (siFUS). siFUS sequence: 5′-UAUAGUUACAAUUACAUAGUCCGACAC-3′ (IDT, DsiRNA). The siRNA was transfected using Lipofectamine 2000 (Invitrogen) as per manufacturer's recommendation. At 48 h post transfection of siRNA, cells were retransfected with pDUP-FUS-E7L plasmid alone or together with EGFP or EGFP-FUS plasmid to rescue the FUS protein levels. At 24 h post re-transfection, cytoplasmic RNA was isolated for analysis of splice variants by RT-PCR, as described above.

### Western blot and quantitative RT-PCR

Rabbit anti-FUS antibody (Bethyl Laboratories, BL1355) or mouse anti-FUS antibody (10F7), rabbit anti-Actin antibody (Sigma, A2066), mouse anti-SF2 (Santa Cruz, SC73026) and mouse anti-hnRNP A1 (ImmuQuest, IQ205) were used for western blot analysis. Western blot was developed using ECL prime reagent (Amersham) and imaged with ChemiDoc MP imaging system (Bio-Rad). The protein band intensity was quantified using the ImageJ program v1.44p (NIH, Bethesda, MD, USA, http://rsbweb.nih.gov/ij/). Actin was used to normalize the loading of protein amount.

The endogenous human *FUS* transcripts were measured by quantitative RT-PCR with qPCR SYBR Green mix (Fermentas), using a real-time PCR system (Bio-Rad, CFX96). Primers were designed to anneal to the 3′ UTR region of the endogenous *FUS* transcript. Primer sequences: 5′-CCAATTCCTGATCACCCAAGGGTTT-3′ (forward), 5′-TGGGCAGGGTAATCTGAACAGGAA-3′ (reverse).

### Splicing modulating antisense oligonucleotide (ASO) assay

2′-O-methyl-oligoribonucleotides with phosphorothioate linkages were synthesized and purified by Trilink Biotechnologies, Inc. (San Diego, CA). FUS-ASOs target the splice junction spanning intron 6 and exon 7. FUS-ASO sequences: 5′-GUCACUUCCGCUGGAGAAGA-3′. Control ASOs (Ctrl-ASOs) are ASOs targeting the *SRA* gene [73]. ASOs alone (final concentration 2 nM, 10 nM and 50 nM) or ASOs together with pDUP-FUS-E7L reporters (0.5 µg) was transfected into HEK293 cells using Lipofectamine 2000 reagent (Invitrogen) as per manufacturer's recommendation. The splicing of exon 7 in the reporter was assessed by RT-PCR, as described above.

#### Accession code

All CLIP-seq data was registered in the Gene Expression Omnibus (accession code: GSE50178).

## Supporting Information

Figure S1 FUS CLIP-seq in HeLa cells. A) Bioinformatics work flowchart to analyze FUS CLIP reads (tags) and determine CLIP clusters. B) Number of FUS CLIP reads in different RNA categories.
(TIF)

Figure S2 Comparison of FUS CLIP-seq RNA targets from various cells and tissues. A) The number of overlapping RNA targets between CLIP-seq data in HeLa cells and previous reports. All different datasets were reanalyzed using the same method (see Method) for the comparison. All the gene symbols were converted to HGNC-approved human gene symbols for the comparison. The percentage was calculated by dividing the number of overlapping genes by the total number of genes in our CLIP-seq data. B) Venn diagram indicates overlapping RNA targets between the CLIP-seq data in HeLa cells (Zhou *et al*.) and the PAR-CLIP data in HEK293 cells (Hoell *et al*.). C) Venn diagram indicates overlapping RNA targets among CLIP-seq data of mouse brains (Ishigaki *et al*.; Rogelj *et al*.; Lagier-Tourenne *et al*.) and neurons (Nakaya *et al*.). D) Venn diagram indicates overlapping RNA targets among CLIP-seq data of HeLa cells, HEK293 cells, mouse brains and neurons.
(TIF)

Figure S3 FUS CLIP clusters associated with alternative splicing events. A) The percentages of FUS-associated alternative events. Using UCSC Known AltEvent database as a reference, a FUS CLIP cluster was considered to be associated with an alternative splicing event if the cluster was within the region covering an alternative exon, or its flanking introns and constitutive exons. The percentage represents the number of FUS-associated alternative events in each category divided by the total number of FUS-associated alternative events. The control was an average percentage of 100 sets of random trials. B) The significance of FUS CLIP clusters associated alternative events. “Total” represents the total number of alternative events in each category in the UCSC Known AltEvent database. “Observed” represents the number of alternative events associated with FUS CLIP clusters. “Expected” represents the average number of alternative events from 100 random trials, as described in A. Z-score shows the significance for the comparison between the observed and the expected.
(TIF)

Figure S4 Consensus RNA motif analysis of FUS CLIP clusters in cassette exons and flanking introns. Sequences of FUS CLIP clusters encompassed by the highest binding peak in the normalized complexity map (5′ splice sites downstream of cassette exons in Figure 1E) were analyzed using the HOMER algorithm to identify possible consensus RNA motifs. Randomized RNA sequences of the same length from the human genome hg19 were used as control.
(TIF)

Figure S5 RNA motif analysis of CLIP tags in *FUS* intron 6 and intron 7. A) Mapping of CLIP tags in the *FUS* intron6-exon7-intron7 region. The graph was generated using the CisGenome program. Blue boxes indicate that sub-regions with highly enriched CLIP tags (over 100 overlapping CLIP tags in the center) were used for *de novo* consensus RNA motif analysis. B) Consensus RNA motif analysis of all CLIP tags within these selected regions in A) using the Homer algorithm. Randomized RNA sequences of the same length from the human genome hg19 were used as control. The top ranked motifs are shown.
(TIF)

Figure S6 FUS protein levels are not affected by cycloheximide (CHX) treatment for 6 h. Western blot analysis of FUS protein levels in four different cells treated with or without 100 µg/ml CHX for 6 h. Bar graphs represent mean ± SEM (n = 3). For each cell line, the CHX treated sample was compared with the untreated sample using student's *t*-tests. “NS” indicates no statistical significance.
(TIF)

Figure S7 Quantification of endogenous *FUS* mRNA levels after exogenous expression of EGFP-FUS. Endogenous *FUS* mRNA levels were quantified by qRT-PCR at 48 h post transfection of EGFP-FUS in HEK293 cells. Primers only anneal to the 3′ UTR of endogenous FUS transcripts but not the EGFP-FUS transcripts. Relative expression was calculated as 2^-ΔΔCt^, using 18s rRNA as a loading control. Bar graphs represent mean ± SEM (n = 3). No statistical significance (student's *t*-test) was observed between cells transfected with EGFP-FUS and untransfected cells (mock).
(TIF)

Figure S8 Endogenous FUS protein is localized in the cytoplasmic aggregates of FUS ΔE15 mutants expressed in mouse motor neuron cells NSC-34. A) Confocal fluorescent microscopy showing the cellular localization of EGFP-FUS mutants in NSC-34 cells. Magnification, 40×. Scale bar, 20 µm. Endogenous FUS protein was detected using anti-FUS antibody (Bethyl, BL1355). DNA in the nucleus was stained with DAPI or NucRed Dead 647. B) Confocal fluorescent microscopy showing the localization of endogenous FUS protein in the cytoplasmic aggregates of EGFP-FUS ΔE15 mutants expressed in NSC-34 cells. Anti-FUS antibody (Bethyl, BL1355) recognizing a C-terminus epitope detected only endogenous FUS protein but not ΔE15 mutants. Magnification, 40×. Scale bar, 20 µm.
(TIF)

Figure S9 ALS-associated FUS mutants are deficient in repressing *FUS* exon 7 in SH-SY5Y cells. RT-PCR analysis of *FUS* exon 7 splice variants in pDUP-FUS-E7L coexpressed with either wildtype (wt) or mutant EGFP-FUS in SH-SY5Y cells. Bar graphs represent mean ± SD (n = 3). EGFP-FUS mutants were compared with EGFP-FUS wildtype protein using student's *t*-test. * *P*≤0.05, ** *P*≤0.01.
(TIF)

Table S1 List of RNA targets identified in FUS CLIP-seq in HeLa cells.
(XLS)

Table S2 Gene Ontology (GO) biological process analysis of overlapping RNA targets of FUS between CLIP-seq datasets in different cells and tissues.
(XLS)

Table S3 List of FUS-associated cassette exons in the complexity map.
(PDF)

Table S4 Gene Ontology (GO) biological process analysis of genes encoding FUS-associated cassette exons.
(PDF)

Table S5 KEGG pathways of genes encoding FUS-associated cassette exons.
(PDF)
