# Supplementary figures and images for: ALS-Associated FUS Mutations Result in Compromised FUS Alternative Splicing and Autoregulation
Source: PLoS Genet. 2013 Oct 31;9(10):e1003895. doi: 10.1371/journal.pgen.1003895 (PMC3814325; doi:10.1371/journal.pgen.1003895)

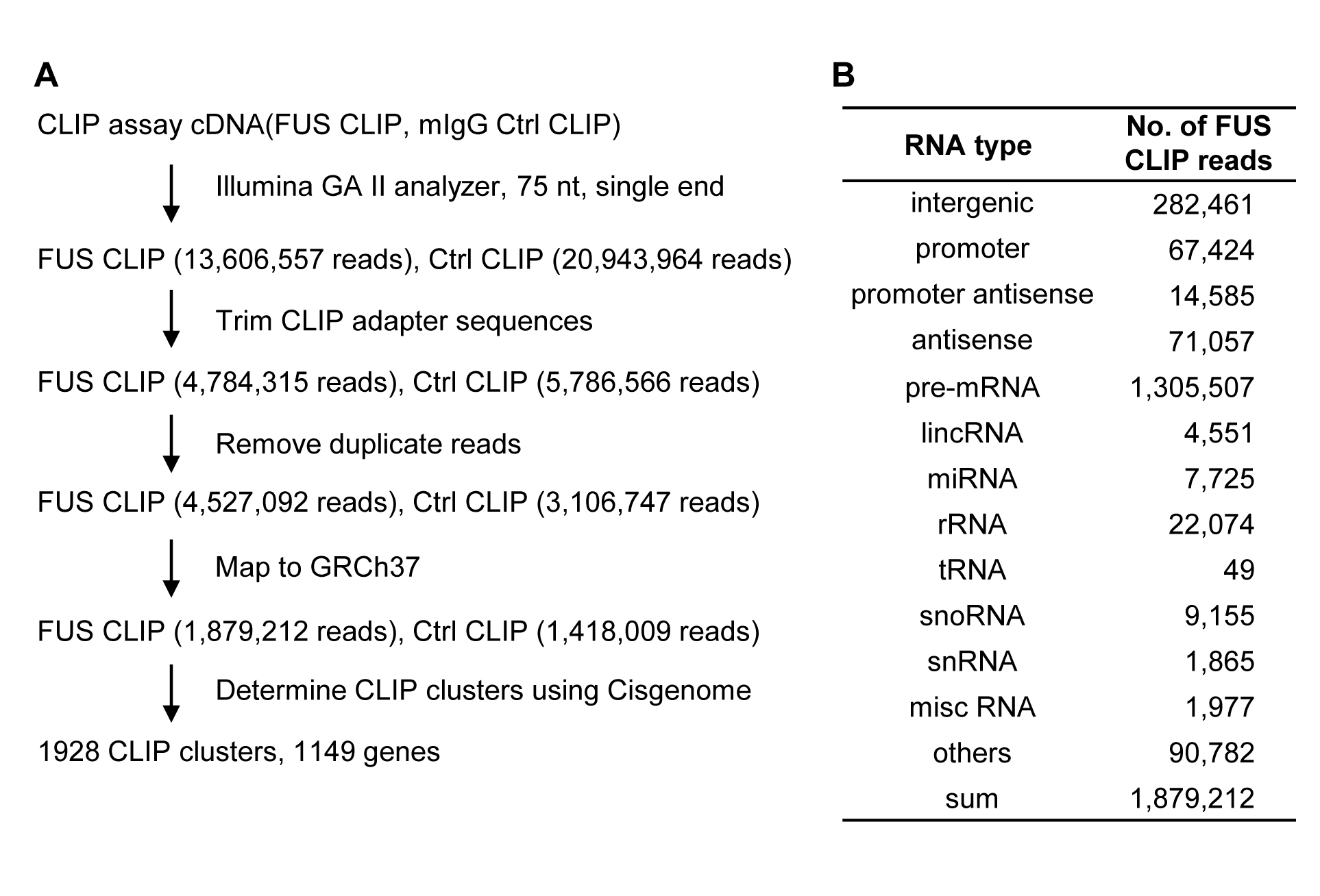

Supplement: Figure S1 — FUS CLIP-seq in HeLa cells. A) Bioinformatics work flowchart to analyze FUS CLIP reads (tags) and determine CLIP clusters. B) Number of FUS CLIP reads in different RNA categories. (TIF) [file pgen.1003895.s001.tif]

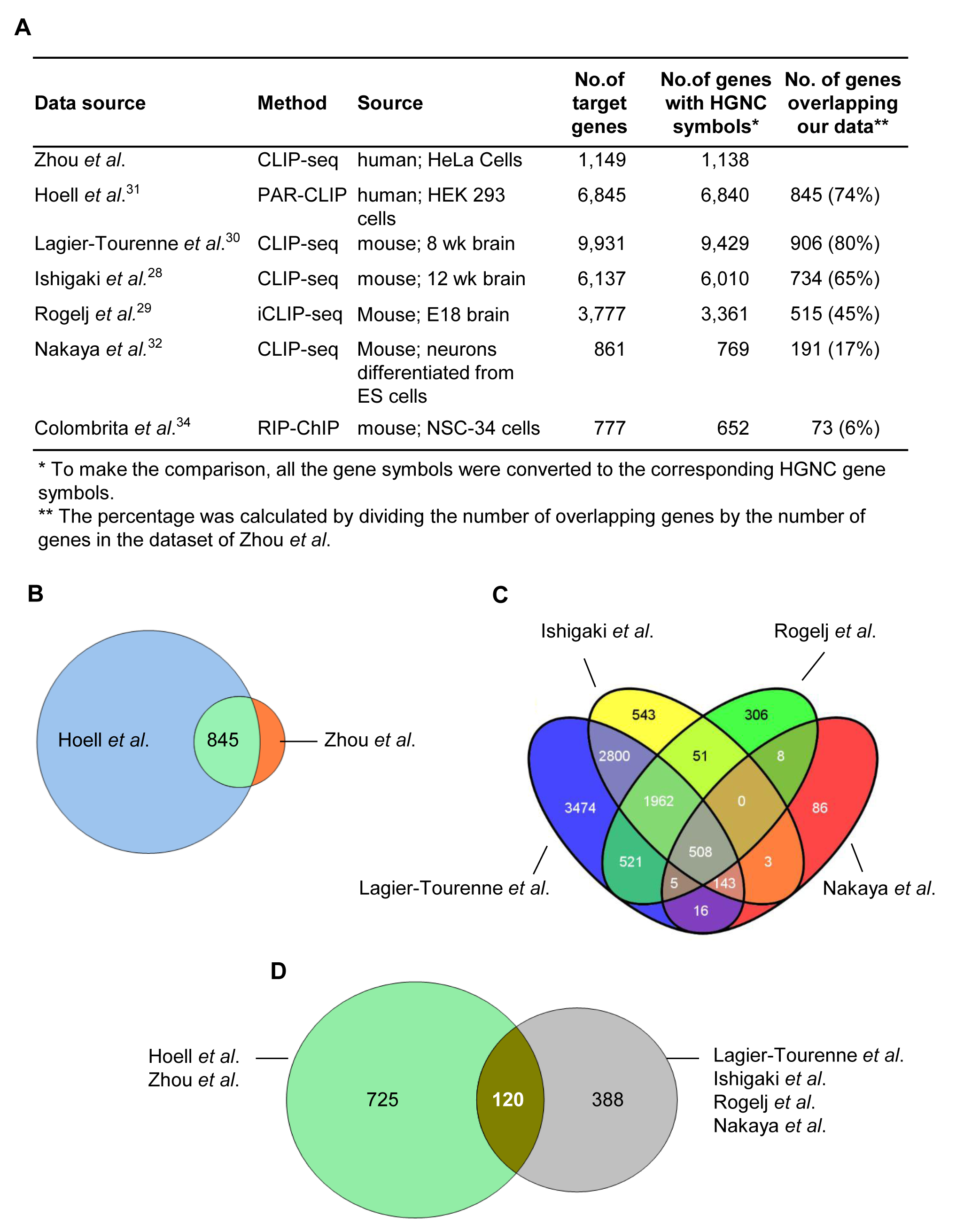

Supplement: Figure S2 — Comparison of FUS CLIP-seq RNA targets from various cells and tissues. A) The number of overlapping RNA targets between CLIP-seq data in HeLa cells and previous reports. All different datasets were reanalyzed using the same method (see Method) for the comparison. All the gene symbols were converted to HGNC-approved human gene symbols for the comparison. The percentage was calculated by dividing the number of overlapping genes by the total number of genes in our CLIP-seq data. B) Venn diagram indicates overlapping RNA targets between the CLIP-seq data in HeLa cells (Zhou et al.) and the PAR-CLIP data in HEK293 cells (Hoell et al.). C) Venn diagram indicates overlapping RNA targets among CLIP-seq data of mouse brains (Ishigaki et al.; Rogelj et al.; Lagier-Tourenne et al.) and neurons (Nakaya et al.). D) Venn diagram indicates overlapping RNA targets among CLIP-seq data of HeLa cells, HEK293 cells, mouse brains and neurons. (TIF) [file pgen.1003895.s002.tif]

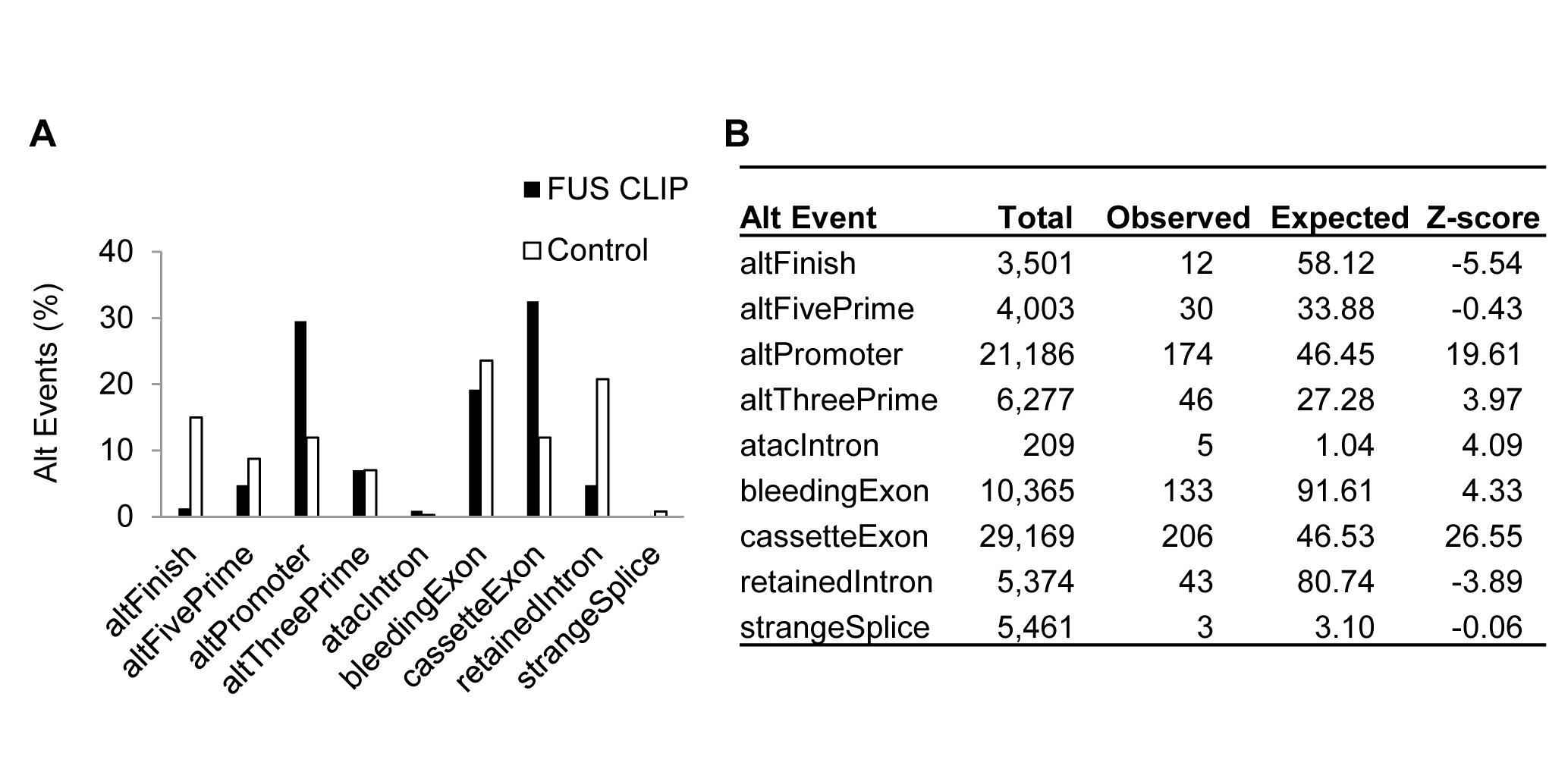

Supplement: Figure S3 — FUS CLIP clusters associated with alternative splicing events. A) The percentages of FUS-associated alternative events. Using UCSC Known AltEvent database as a reference, a FUS CLIP cluster was considered to be associated with an alternative splicing event if the cluster was within the region covering an alternative exon, or its flanking introns and constitutive exons. The percentage represents the number of FUS-associated alternative events in each category divided by the total number of FUS-associated alternative events. The control was an average percentage of 100 sets of random trials. B) The significance of FUS CLIP clusters associated alternative events. “Total” represents the total number of alternative events in each category in the UCSC Known AltEvent database. “Observed” represents the number of alternative events associated with FUS CLIP clusters. “Expected” represents the average number of alternative events from 100 random trials, as described in A. Z-score shows the significance for the comparison between the observed and the expected. (TIF) [file pgen.1003895.s003.tif]

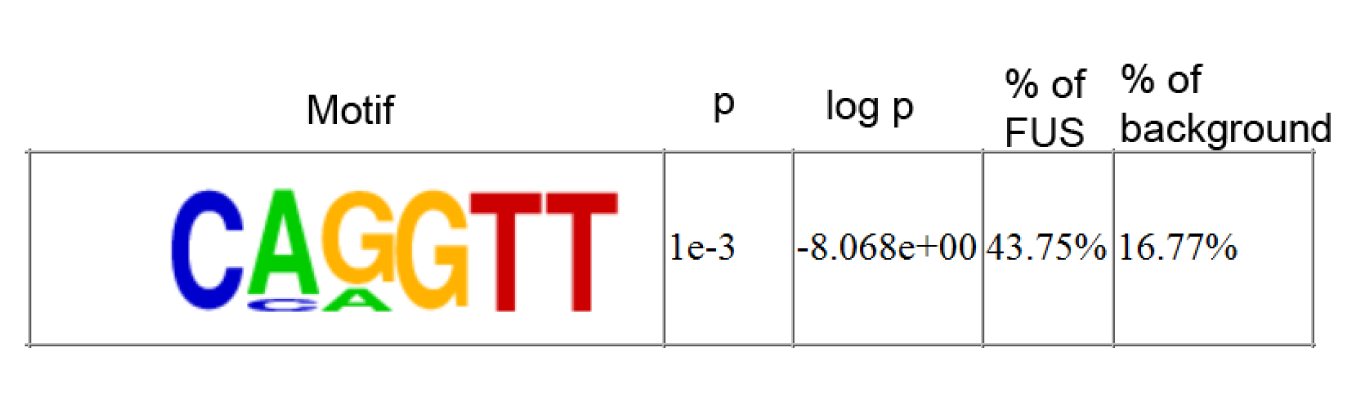

Supplement: Figure S4 — Consensus RNA motif analysis of FUS CLIP clusters in cassette exons and flanking introns. Sequences of FUS CLIP clusters encompassed by the highest binding peak in the normalized complexity map (5′ splice sites downstream of cassette exons in Figure 1E) were analyzed using the HOMER algorithm to identify possible consensus RNA motifs. Randomized RNA sequences of the same length from the human genome hg19 were used as control. (TIF) [file pgen.1003895.s004.tif]

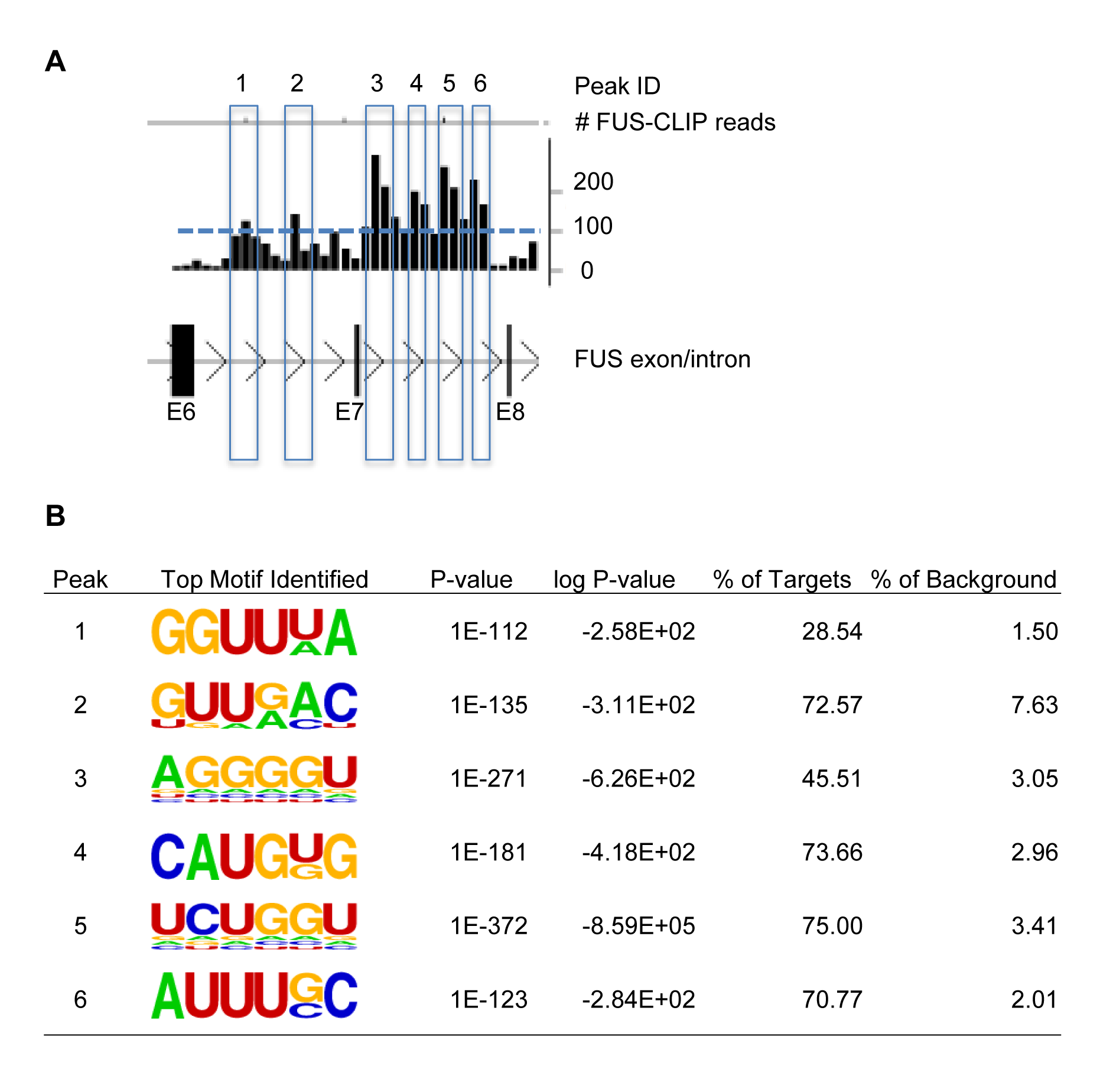

Supplement: Figure S5 — RNA motif analysis of CLIP tags in FUS intron 6 and intron 7. A) Mapping of CLIP tags in the FUS intron6-exon7-intron7 region. The graph was generated using the CisGenome program. Blue boxes indicate that sub-regions with highly enriched CLIP tags (over 100 overlapping CLIP tags in the center) were used for de novo consensus RNA motif analysis. B) Consensus RNA motif analysis of all CLIP tags within these selected regions in A) using the Homer algorithm. Randomized RNA sequences of the same length from the human genome hg19 were used as control. The top ranked motifs are shown. (TIF) [file pgen.1003895.s005.tif]

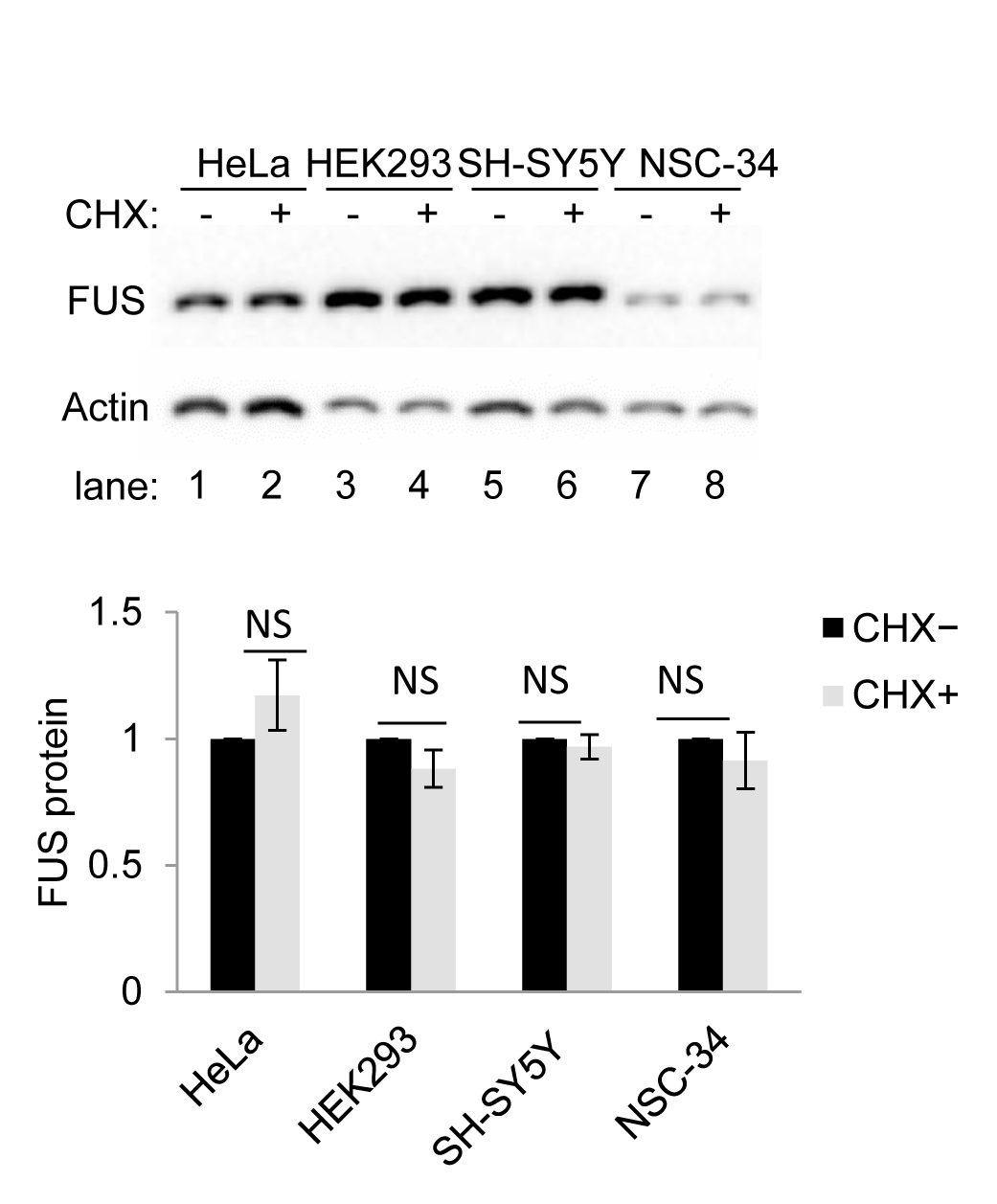

Supplement: Figure S6 — FUS protein levels are not affected by cycloheximide (CHX) treatment for 6 h. Western blot analysis of FUS protein levels in four different cells treated with or without 100 µg/ml CHX for 6 h. Bar graphs represent mean ± SEM (n = 3). For each cell line, the CHX treated sample was compared with the untreated sample using student's t-tests. “NS” indicates no statistical significance. (TIF) [file pgen.1003895.s006.tif]

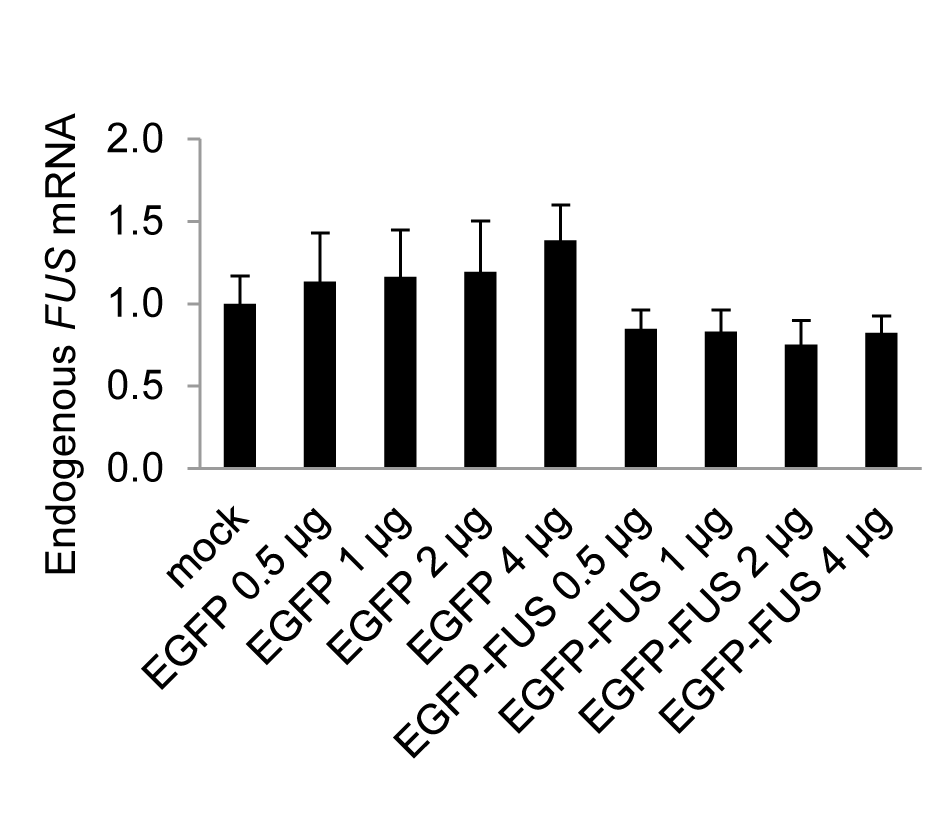

Supplement: Figure S7 — Quantification of endogenous FUS mRNA levels after exogenous expression of EGFP-FUS. Endogenous FUS mRNA levels were quantified by qRT-PCR at 48 h post transfection of EGFP-FUS in HEK293 cells. Primers only anneal to the 3′ UTR of endogenous FUS transcripts but not the EGFP-FUS transcripts. Relative expression was calculated as 2-ΔΔCt, using 18s rRNA as a loading control. Bar graphs represent mean ± SEM (n = 3). No statistical significance (student's t-test) was observed between cells transfected with EGFP-FUS and untransfected cells (mock). (TIF) [file pgen.1003895.s007.tif]

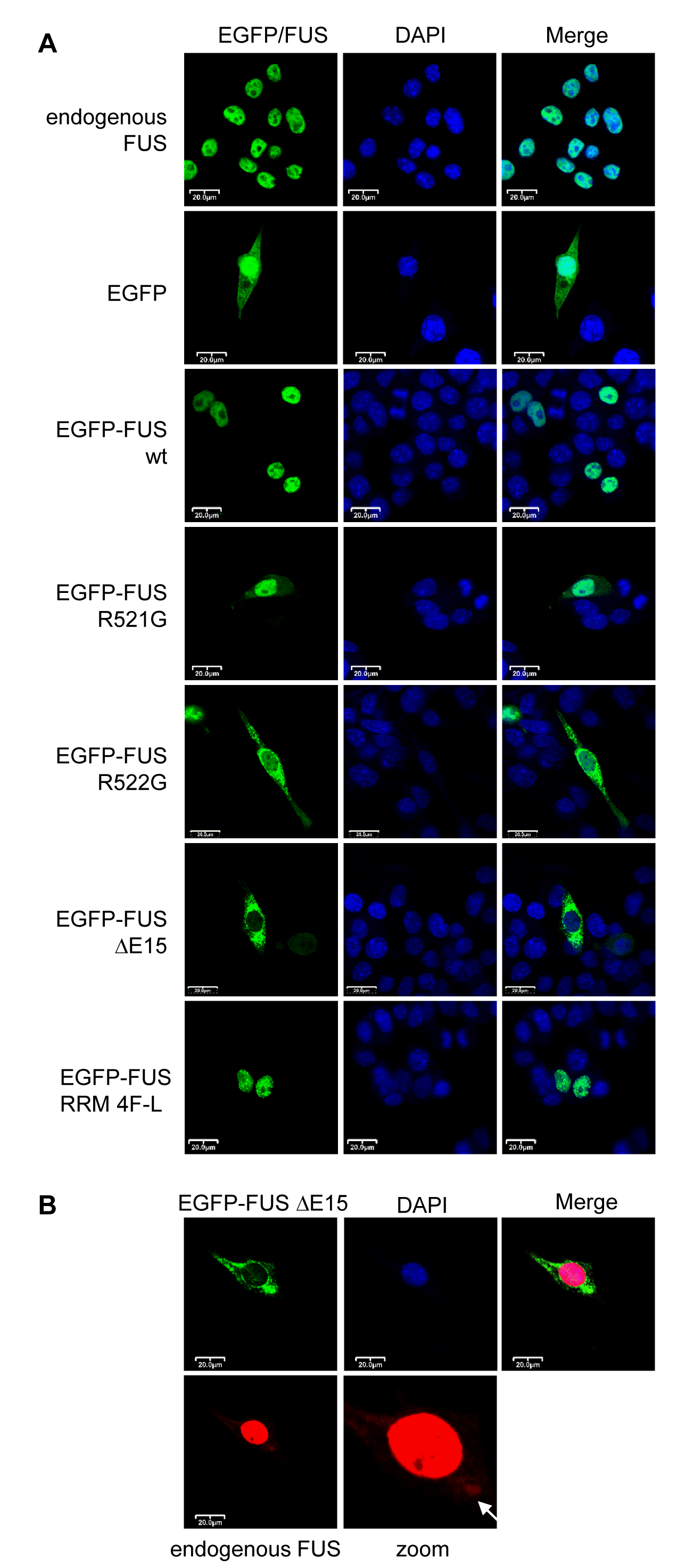

Supplement: Figure S8 — Endogenous FUS protein is localized in the cytoplasmic aggregates of FUS ΔE15 mutants expressed in mouse motor neuron cells NSC-34. A) Confocal fluorescent microscopy showing the cellular localization of EGFP-FUS mutants in NSC-34 cells. Magnification, 40×. Scale bar, 20 µm. Endogenous FUS protein was detected using anti-FUS antibody (Bethyl, BL1355). DNA in the nucleus was stained with DAPI or NucRed Dead 647. B) Confocal fluorescent microscopy showing the localization of endogenous FUS protein in the cytoplasmic aggregates of EGFP-FUS ΔE15 mutants expressed in NSC-34 cells. Anti-FUS antibody (Bethyl, BL1355) recognizing a C-terminus epitope detected only endogenous FUS protein but not ΔE15 mutants. Magnification, 40×. Scale bar, 20 µm. (TIF) [file pgen.1003895.s008.tif]

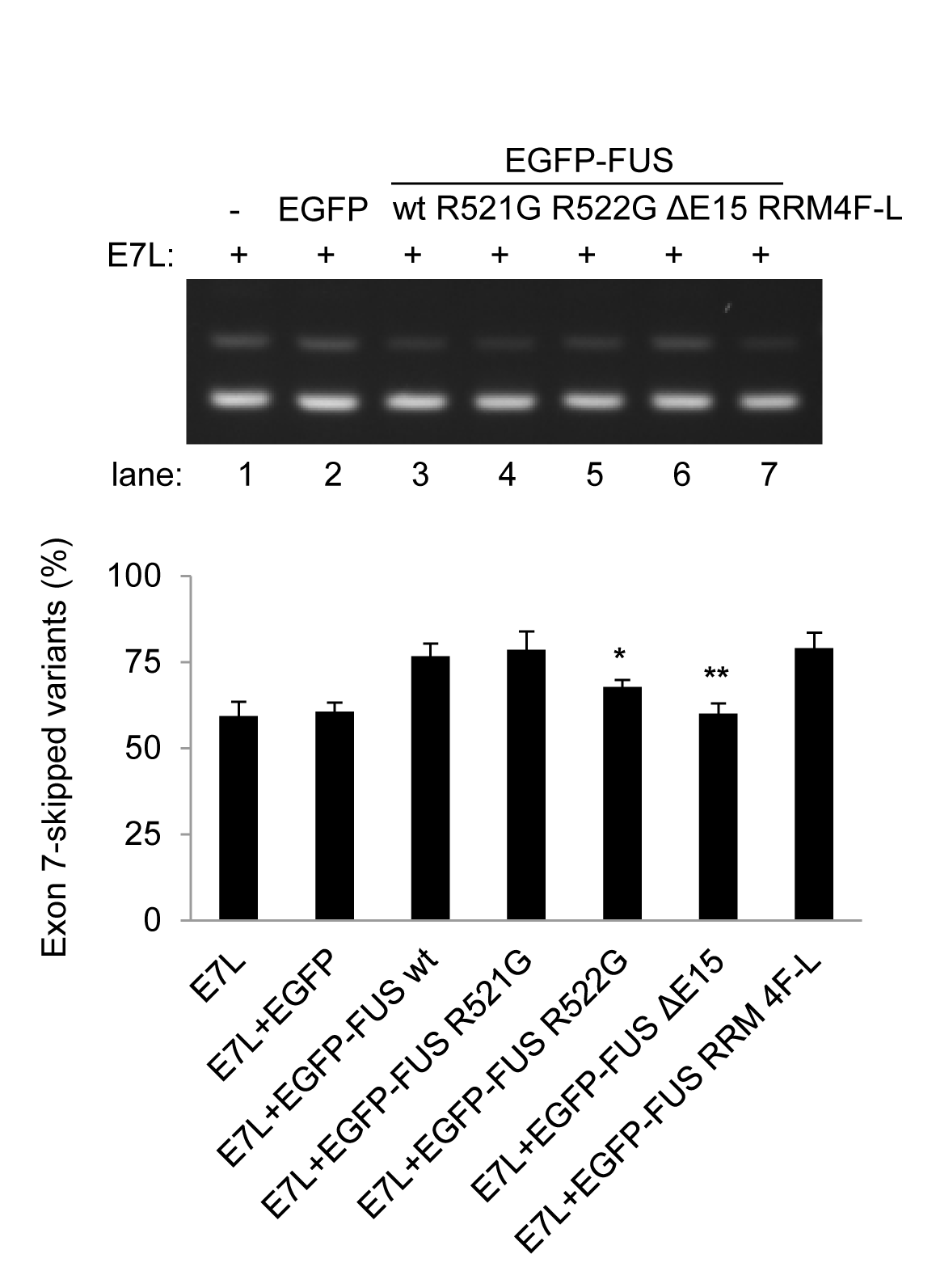

Supplement: Figure S9 — ALS-associated FUS mutants are deficient in repressing FUS exon 7 in SH-SY5Y cells. RT-PCR analysis of FUS exon 7 splice variants in pDUP-FUS-E7L coexpressed with either wildtype (wt) or mutant EGFP-FUS in SH-SY5Y cells. Bar graphs represent mean ± SD (n = 3). EGFP-FUS mutants were compared with EGFP-FUS wildtype protein using student's t-test. * P≤0.05, ** P≤0.01. (TIF) [file pgen.1003895.s009.tif]
