# Supplementary material for: ALS-Associated FUS Mutations Result in Compromised FUS Alternative Splicing and Autoregulation
Source: PLoS Genet. 2013 Oct 31;9(10):e1003895. doi: 10.1371/journal.pgen.1003895 (PMC3814325; doi:10.1371/journal.pgen.1003895)
Supplement: Table S3 — List of FUS-associated cassette exons in the complexity map. (PDF) [file pgen.1003895.s012.pdf]

Table S3. List of FUS-associated cassette exons in the complexity map

Cluster Rank	GeneSymbol	RefSeqID	Chr	Strand	ExonStart	ExonEnd
7	FUS	NM_001170634	chr16	+	31198122	31198157
20	FUS	NM_001170634	chr16	+	31200443	31200547
44	CD55	NM_000574	chr1	+	207497903	207498095
56	VAPB	NM_004738	chr20	+	57015962	57016139
74	TBC1D14	NM_001113363	chr4	+	7011603	7011675
91	PBX1	NM_002585	chr1	+	164532474	164532548
114	PDPK1	NM_002613	chr16	+	2616356	2616454
130	ATP2A2	NM_170665	chr12	+	110784005	110788897
148	TCF12	NM_207038	chr15	+	57355947	57356021
177	OGDH	NM_001003941	chr7	+	44684925	44685117
219	PDPK1	NM_002613	chr16	+	2647123	2647276
224	C11orf49	NM_001003676	chr11	+	47073938	47074069
225	MXI1	NM_005962	chr10	+	112004585	112004615
249	TACC2	NM_206861	chr10	+	123903086	123903221
260	GLI2	NM_005270	chr2	+	121684936	121685042
266	SYNE2	NM_182914	chr14	+	64656788	64656955
289	ZNF182	NM_006962	chrX	-	47847923	47848017
301	WDR25	NM_024515	chr14	+	100934357	100934505
336	SAP30BP	NM_013260	chr17	+	73667895	73667943
358	PDE4B	NM_001037340	chr1	+	66384279	66384518
402	ACTN4	NM_004924	chr19	+	39191239	39191354
415	IGF1R	NM_000875	chr15	+	99478052	99478282
429	MCM8	NM_032485	chr20	+	5953701	5953842
453	CDADC1	NM_030911	chr13	+	49833481	49833659
494	PAN3	NM_175854	chr13	+	28771321	28771483

508	CASP8	NM_033358	chr2	+	202131183	202131514
512	EFCAB2	NR_026588	chr1	+	245180543	245180628
515	CUX1	NM_181552	chr7	+	101671377	101671425
526	LRRC61	NM_001142928	chr7	+	150022929	150023099
538	PIK3C3	NM_002647	chr18	+	39537534	39537723
556	NVL	NM_206840	chr1	-	224455735	224455835
563	CACNA2D3	NM_018398	chr3	+	54850879	54850897
568	TNRC6A	NM_014494	chr16	+	24804793	24804970
578	RBM6	NM_005777	chr3	+	50085677	50085752
582	SPIRE1	NM_001128627	chr18	-	12546672	12546903
597	PDLIM5	NM_001011516	chr4	+	95444874	95445026
598	LIMCH1	NM_001112717	chr4	+	41496552	41496623
628	OSBPL6	NM_145739	chr2	+	179236852	179236960
682	RPTOR	NM_020761	chr17	+	78617527	78617610
740	MED30	NM_080651	chr8	+	118542961	118543066
772	DNM1L	NM_012062	chr12	+	32891197	32891230
798	SWAP70	NM_015055	chr11	+	9735012	9735186
799	TCF7L2	NM_001146284	chr10	+	114799783	114799885
811	PSMF1	NM_006814	chr20	+	1115763	1115949
824	CDKL5	NM_001037343	chrX	+	18460494	18460543
825	ADAMTS13	NM_139027	chr9	+	136308496	136308682
858	ARHGEF10L	NM_001011722	chr1	+	17961042	17961057
879	TBC1D19	NM_018317	chr4	+	26622234	26622310
955	PSMG4	NM_001128592	chr6	+	3264442	3264559
965	MEIS1	NM_002398	chr2	+	66739280	66739426
1004	EYA4	NM_172103	chr6	+	133777693	133777786
1025	IKZF2	NM_016260	chr2	-	213921556	213921823

1036	CEP192	NM_032142	chr18	+	13113584	13113704
1048	ATE1	NM_001001976	chr10	-	123629520	123629553
1119	KIF13B	NM_015254	chr8	-	29053703	29053716
1137	GPD2	NM_000408	chr2	+	157352555	157352727
1141	HNRNPUL1	NM_007040	chr19	+	41787067	41787180
1150	TOMM20L	NM_207377	chr14	+	58869397	58869479
1154	TMTC2	NM_152588	chr12	+	83250788	83251359
1269	EPB41L1	NM_177996	chr20	+	34700347	34700402
1295	SNAP47	NM_053052	chr1	+	227935392	227935934
1299	WDR3	NM_006784	chr1	+	118501532	118501626
1307	UBAP2	NM_018449	chr9	-	33956076	33956144
1312	MAPK14	NM_139013	chr6	+	36070347	36070535
1317	SORBS2	NM_001145675	chr4	-	186560030	186560189
1349	UBE2E2	NM_152653	chr3	+	23541098	23541231
1464	MED15	NM_015889	chr22	+	20909222	20909435
1477	PAPPA	NM_002581	chr9	+	118973917	118974211
1508	MYPN	NM_032578	chr10	+	69881194	69882097
1535	MAPK14	NM_139013	chr6	+	36063763	36063843
1563	TIMM50	NM_001001563	chr19	+	39976183	39976242
1579	LDLRAD3	NM_174902	chr11	+	36057652	36057799
1581	PARD3	NM_019619	chr10	-	34739244	34739376
1625	LIMCH1	NM_001112719	chr4	+	41640948	41640984
1645	CRTC3	NM_001042574	chr15	+	91136867	91136987
1661	PAPPA	NM_002581	chr9	+	118969734	118969880
1671	NUPL2	NM_007342	chr7	+	23235457	23235534
1674	SLC22A3	NM_021977	chr6	+	160857811	160857909
1677	RUNX2	NM_004348	chr6	+	45512953	45513019

1679	CDYL	NM_001143971	chr6	+	4891946	4892613
1717	TMUB2	NM_177441	chr17	+	42266389	42266956
1786	SOLH	NM_005632	chr16	+	586028	586142
1801	TBL1X	NM_005647	chrX	+	9502939	9503074
1837	KIRREL	NM_018240	chr1	+	158045902	158046052
1870	ATG7	NM_001144912	chr3	+	11468277	11468400
1883	POLR1B	NM_019014	chr2	+	113316869	113317151
1897	FUT8	NM_178154	chr14	+	65922338	65922436
