# Supplementary material for: ALS-Associated FUS Mutations Result in Compromised FUS Alternative Splicing and Autoregulation
Source: PLoS Genet. 2013 Oct 31;9(10):e1003895. doi: 10.1371/journal.pgen.1003895 (PMC3814325; doi:10.1371/journal.pgen.1003895)
Supplement: Table S4 — Gene Ontology (GO) biological process analysis of genes encoding FUS-associated cassette exons. (PDF) [file pgen.1003895.s013.pdf]

Table S4. Gene Ontology (GO) biological process analysis of genes encoding FUS-associated cassette exons

**Annotation Cluster 1***	**Enrichment Score: 2.677720623890005**		
**Category**	**Term**	**%**	**PValue**
GOTERM_BP_FAT	GO:0045944~positive regulation of transcription from RNA polymerase II promoter	7.73	1.92E-04
GOTERM_BP_FAT	GO:0010604~positive regulation of macromolecule metabolic process	11.60	1.60E-03
GOTERM_BP_FAT	GO:0045935~positive regulation of nucleobase, nucleoside, nucleotide and nucleic acid metabolic process	9.39	1.67E-03
GOTERM_BP_FAT	GO:0045941~positive regulation of transcription	8.84	1.81E-03
GOTERM_BP_FAT	GO:0010557~positive regulation of macromolecule biosynthetic process	9.39	2.04E-03
GOTERM_BP_FAT	GO:0010628~positive regulation of gene expression	8.84	2.07E-03
GOTERM_BP_FAT	GO:0051173~positive regulation of nitrogen compound metabolic process	9.39	2.30E-03
GOTERM_BP_FAT	GO:0031328~positive regulation of cellular biosynthetic process	9.39	2.66E-03
GOTERM_BP_FAT	GO:0045893~positive regulation of transcription, DNA-dependent	7.73	2.90E-03
GOTERM_BP_FAT	GO:0009891~positive regulation of biosynthetic process	9.39	2.98E-03
GOTERM_BP_FAT	GO:0051254~positive regulation of RNA metabolic process	7.73	3.15E-03
GOTERM_BP_FAT	GO:0006357~regulation of transcription from RNA polymerase II promoter	9.39	1.13E-02
**Annotation Cluster 2**	**Enrichment Score: 1.8741283471568786**		
**Category**	**Term**	**%**	**PValue**
GOTERM_BP_FAT	GO:0010629~negative regulation of gene expression	8.29	3.86E-03
GOTERM_BP_FAT	GO:0010558~negative regulation of macromolecule biosynthetic process	8.29	6.78E-03

GOTERM_BP_FAT	GO:0031327~negative regulation of cellular biosynthetic process	8.29	7.24E-03
GOTERM_BP_FAT	GO:0009890~negative regulation of biosynthetic process	8.29	8.43E-03
GOTERM_BP_FAT	GO:0016481~negative regulation of transcription	7.18	1.09E-02
GOTERM_BP_FAT	GO:0010605~negative regulation of macromolecule metabolic process	9.39	2.22E-02
GOTERM_BP_FAT	GO:0045934~negative regulation of nucleobase, nucleoside, nucleotide and nucleic acid metabolic process	7.18	2.40E-02
GOTERM_BP_FAT	GO:0051172~negative regulation of nitrogen compound metabolic process	7.18	2.59E-02
GOTERM_BP_FAT	GO:0045892~negative regulation of transcription, DNA-dependent	5.52	2.59E-02
GOTERM_BP_FAT	GO:0051253~negative regulation of RNA metabolic process	5.52	2.90E-02
**Annotation Cluster 3**	**Enrichment Score: 1.5872937944810788**		
**Category**	**Term**	**%**	**PValue**
GOTERM_BP_FAT	GO:0060284~regulation of cell development	3.87	1.72E-02
GOTERM_BP_FAT	GO:0050767~regulation of neurogenesis	3.31	2.08E-02
GOTERM_BP_FAT	GO:0051960~regulation of nervous system development	3.31	3.32E-02
GOTERM_BP_FAT	GO:0045664~regulation of neuron differentiation	2.76	3.77E-02

* GO functional annotation clustering was performed using DAVID Bioinformatics Resources 6.7 (http://david.abcc.ncifcrf.gov/).
