# Supplementary material for: ALS-Associated FUS Mutations Result in Compromised FUS Alternative Splicing and Autoregulation
Source: PLoS Genet. 2013 Oct 31;9(10):e1003895. doi: 10.1371/journal.pgen.1003895 (PMC3814325; doi:10.1371/journal.pgen.1003895)
Supplement: Table S5 — KEGG pathways of genes encoding FUS-associated cassette exons. (PDF) [file pgen.1003895.s014.pdf]

Table S5. KEGG pathways of genes encoding FUS-associated cassette exons

Category*	Term	%	PValue
KEGG_PATHWAY	hsa04310:Wnt signaling pathway	3.87	1.33E-02
KEGG_PATHWAY	hsa04520:Adherens junction	2.76	3.33E-02
KEGG_PATHWAY	hsa04330:Notch signaling pathway	2.21	3.58E-02

* Analysis of KEGG pathways was performed using DAVID Bioinformatics Resources 6.7 (http://david.abcc.ncifcrf.gov/).
